# The Histone‐Lysine N‐Methyltransferase (KMT2) Family in Health and Disease

**DOI:** 10.1002/mco2.70728

**Published:** 2026-04-09

**Authors:** Qiu Wang, Zunjie Bo, Ya Zhang, Yun Chen, Zhiyu Wang, Shuangmei Tong, Ajing Xu, Jian Zhang, Yan Liu

**Affiliations:** ^1^ Department of Clinical Pharmacy Xinhua Hospital Affiliated to Shanghai Jiao Tong University School of Medicine Shanghai China; ^2^ Dalian Medical University Dalian China; ^3^ Department of Clinical Pharmacy Shanghai General Hospital, Shanghai Jiao Tong University School of Medicine Shanghai China

**Keywords:** disease, epigenetics, KMT2 family, therapy

## Abstract

The histone‐lysine N‐methyltransferase (KMT2) family is a central epigenetic regulator whose dysfunction drives diverse human diseases through distinct molecular mechanisms. In acute leukemias, KMT2A rearrangements aberrantly recruit transcriptional cofactors, activating oncogenic gene programs; in solid tumors, loss‐of‐function mutations in KMT2C/D disrupt enhancer‐mediated regulatory networks, compromising cellular identity and genome stability; in neurodevelopmental disorders, germline haploinsufficiency of KMT2A/B/D impairs developmental epigenetic programming. Despite increasingly comprehensive understanding of the pathogenic mechanisms involving KMT2 family members, a unified framework translating these molecular insights into effective, subtype‐specific therapeutic strategies has been lacking. This review comprehensively deconstructs these pathogenic pathways and explores how mechanistic insights are being translated into novel therapeutic strategies, including direct targeting of oncogenic transcriptional complexes,  exploiting vulnerabilities from tumor suppressor loss, and modulating the tumor immune microenvironment. We systematically synthesize recent clinical advances, from small‐molecule inhibitors against protein–protein interactions (e.g., menin–KMT2A), to targeted degraders (PROTACs), epigenetic readers/writers inhibitors (e.g., BET, LSD1, DOT1L), and rational combination regimens with chemotherapy or immunotherapy. By integrating the biological characteristics of KMT2 with translational medicine and clinical evidence, this study provides a framework for advancing precision medicine approaches based on the molecular subtypes driven by KMT2.

## Graphical abstract

This graphical abstract clarifies KMT2 family dysregulation and disease associations: its member mutations cause epigenetic imbalance and abnormal cell development; Menin/WDR5 inhibitors and other targeted therapies block aberrant epigenetic transcriptional programs, and combined synergistic therapies boost therapeutic efficacy.

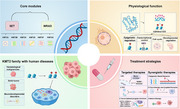

## 1 Introduction

Epigenetic regulation is a core mechanism that maintains the diversity of cell identities and functions in multicellular organisms [1]. It precisely regulates chromatin states and gene expression patterns, enabling cells with the same genome to differentiate into various types with distinct morphologies and functions [2]. Among them, histone modification is a crucial part of this regulatory system, dynamically influencing the structure and function of chromatin [3]. The methylation modification of lysine at position 4 of histone H3, as a marker of active transcriptional regions (especially promoters and enhancers), plays a central role in gene activation [4]. This modification is primarily carried out by “writer” complexes, mainly the COMPASS‐like complexes formed by the histone‐lysine N‐methyltransferase (KMT2) family [5]. These multisubunit complexes catalyze the monomethylation, dimethylation and trimethylation of H3K4 [6], regulating chromatin accessibility, transcription factor recruitment, and the activity of RNA polymerase II [7], thereby playing a central role in transcriptional regulation. Abnormalities in the function of the KMT2 family directly lead to a disorder in the H3K4 methylation landscape, disrupting the normal gene expression program, and are thus closely related to the pathological processes of various human diseases [6, 8].

The KMT2 family is highly conserved during evolution. Its homologues exist from yeast to mammals. Among its members, there is both redundancy in structure and function, as well as lineage‐specificity [9]. The human KMT2 family includes KMT2A to KMT2G (also known as MLL1‐4, SETD1A/B), which can be further classified into different subgroups based on sequence homology, complex composition, and catalytic products [6]. Members of the KMT2 family need to achieve optimal enzymatic activity and genomic localization through multisubunit complexes. The unique subunit composition of each complex not only determines its catalytic activity, substrate specificity, and genomic localization, but also confers its functional specificity in specific biological contexts [8]. Therefore, systematically classifying and structuring the KMT2 family, and deeply analyzing the molecular assembly mechanism of its complexes, is the foundation for understanding its complex functions in both physiological and pathological conditions.

The application of modern omics technologies in recent years has led to a deep transformation in how people perceive the KMT2 family's impact on human health and diseases [10, 11]. Early studies focused on KMT2A gene rearrangement‐driven acute leukemia, revealing the direct driving role of epigenetic dysregulation in hematological malignancies [12, 13, 14]. Extensive genomic data have subsequently shown that mutations in the KMT2 family, particularly KMT2C and KMT2D, represent some of the most frequent epigenetic changes in various solid tumors, including lung cancer and pancreatic ductal adenocarcinoma (PDAC) [7, 15], breast cancer [16, 17], indicating that these proteins play extensive and crucial roles in maintaining cellular homeostasis and inhibiting tumor formation. Additionally, mutations in members such as KMT2A, KMT2B, KMT2C, and KMT2D have been observed. It has been clearly identified as the cause of a series of developmental disorders, such as Wiedemann–Steiner syndrome (WSS), Kabuki syndrome (KS), and dystonia, highlighting the indispensable role of this family in embryonic development and the functioning of the nervous system [18, 19, 20]. This review presents a detailed and methodical analysis of the KMT2 family, integrating its latest advances in normal development and homeostasis maintenance, discussing its multifaceted roles in diseases such as cancer and neurodevelopmental disorders (NDDs), and emphasizing the development, application, and therapeutic prospects of small‐molecule inhibitors targeting KMT2 complexes and related regulators. We hope that our review will help understand the transformation logic from epigenetic mechanisms to disease phenotypes and will also provide key theoretical basis and direction guidance for the development of precise diagnosis and targeted treatment based on epigenetic regulation.

## 2 Structure and Functions of the KMT2 Family

To understand the diverse roles of the KMT2 family in development and disease, it is essential to first examine the structural organization and molecular assembly of its members. KMT2 proteins function as part of multisubunit complexes that catalyze histone H3 lysine 4 methylation, a key epigenetic mark associated with gene activation. This section provides an overview of the conserved architecture of KMT2 complexes, their core and accessory subunits, and the structural basis for their chromatin‐targeting and catalytic activities.

### 2.1 Architecture and Assembly of KMT2 Complexes

**FIGURE 1:**
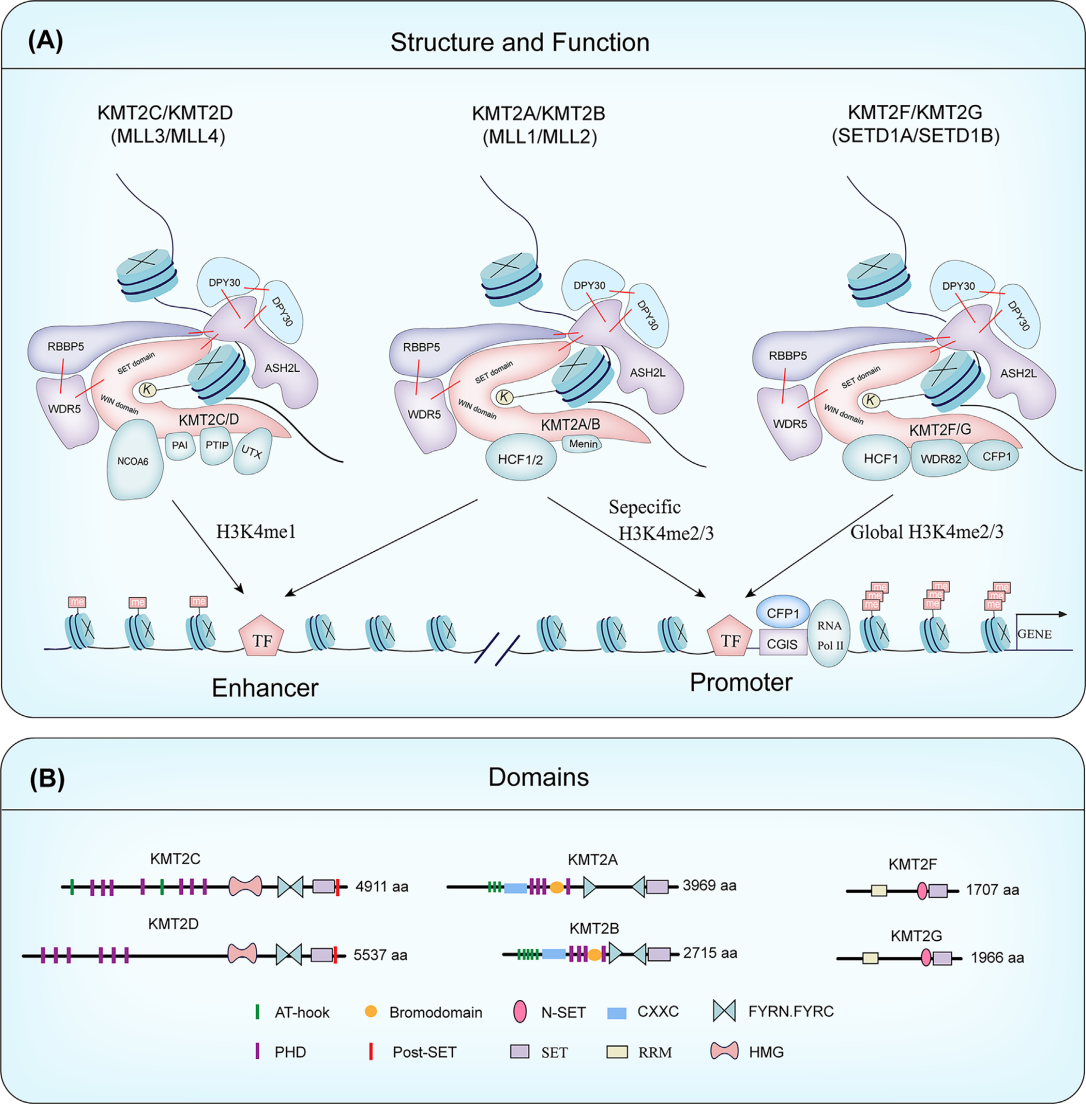
KMT2 histone methyltransferase complexes: structure, function, and domains. (A) Structural organization of distinct KMT2 complexes, their intersubunit interactions (indicated by red lines), and their functional distribution at enhancers or transcription start sites. (B) Domain organization and protein length of human KMT2 family members. *Abbreviations*: AT‐hook, adenosine–thymidine‐hook; CXXC, zinc finger‐CXXC domain; FYRN/C, phenylalanine and tyrosine‐rich region (N‐ and C‐terminal); N‐SET, N‐terminal of SET; PHD, plant homeodomain; Post‐SET, C‐terminal of SET; SET, Su(var)3‐9, enhancer‐of‐zeste and trithorax; RRM, RNA recognition motive; HMG, high mobility group.

In order to deeply understand the diverse roles of the above‐mentioned KMT2 family in diseases, we first need to systematically analyze the structural basis and physiological functions of its protein complexes. The KMT2 family enzymes function as large protein complexes, with their core composed of the SET domain of the catalytic subunit and the conserved WRAD scaffold module (WDR5, RBBP5, ASH2L, DPY30), which forms the structural basis for efficient H3K4 methylation. The overall assembly, nucleosome binding, and catalytic activity of the complex are precisely regulated by a series of specific auxiliary subunits. These subunits guide different complexes to target specific genomic regions such as promoters or enhancers by recognizing chromatin signals or mediating protein–protein interactions (PPIs), thereby performing differentiated transcriptional regulatory functions. The elucidation of the structure and assembly mechanism of the KMT2 complex is central to understanding its physiological functions and the pathogenesis of related diseases. Figure 1 illustrates the structures of the various members of the KMT2 family.

#### 2.1.1 Subunit Composition of KMT2 Enzymes

In mammalian cells, KMT2 family methyltransferases do not function in a free form but exist as multisubunit COMPASS or COMPASS‐like complexes. Their H3K4 methylation activity relies on the coordinated action of a set of highly conserved core subunits and complex‐specific accessory subunits. Although different KMT2 complexes exhibit significant differences in genomic targeting and function, the composition of their core subunits is highly evolutionarily conserved, serving as the foundation for maintaining catalytic activity and chromatin‐binding capacity [6, 21]. This includes the conserved interactions between the common core subunits of the KMT2 complex assembly: the SET module and the WRAD core subunits (WDR5, RBBP5, ASH2L, DPY30). In most KMT2 members, the assembly of WRAD not only stabilizes the modular conformation but also elevates the weak activity of the isolated SET domain to an efficient state capable of achieving physiologically relevant multistate H3K4 methylation [22, 23, 24].

WDR5 is one of the early‐identified core subunits in COMPASS/COMPASS‐like complexes. Its WD40 repeat domain mediates multiple PPIs and plays a key structural role within H3K4 methylation complexes [25]. As a bridging molecule, WDR5 connects the catalytic subunit with the RBBP5–ASH2L module, facilitating correct assembly and stabilizing the conformation of the core complex [26]. However, the degree of dependence on WDR5 varies significantly among different KMT2 family members. In vitro reconstitution studies show that, except for KMT2C, the catalytic efficiency of most KMT2 core complexes relies more heavily on the complete WRAD components, including WDR5 [27, 28]. Among them, the catalytic activity of KMT2A is more dependent on WDR5‐mediated assembly. In contrast, KMT2C can still form an active complex with RBBP5–ASH2L–DPY30 in the absence of WDR5, and under specific in vitro conditions, the addition of WDR5 can even inhibit its H3K4 monomethylation activity. In cellular and mouse models, WDR5 depletion typically leads to a marked decrease in global H3K4 methylation levels (especially H3K4me3), whereas individual depletion of KMT2A does not necessarily cause similar global changes. This suggests that WDR5 likely maintains overall H3K4 methylation status indirectly by participating in various COMPASS and COMPASS‐like complexes [29]. Furthermore, WDR5 can also bind to various protein factors and RNAs (including lncRNAs), participating in chromatin targeting and transcriptional regulation, further supporting its multifunctional role in epigenetic regulation [1, 30].

RBBP5 and ASH2L form a tightly coordinated functional unit within the core COMPASS/COMPASS‐like complex, widely regarded as one of the key modules driving catalytic enhancement. Mechanistically, RBBP5–ASH2L couples with specific interfaces of the KMT2 catalytic SET region, promoting core complex assembly and significantly increasing H3K4 methylation efficiency, particularly facilitating higher‐order methylation conversions. This module constitutes a key structural unit coupled with the SET catalytic module and participates in forming the catalytically active conformation through multiple contact points. In the KMT2A system, internal structural interactions within RBBP5 (interactions between the WD40 domain and the C‐terminal region) can promote a more compact complex conformation and influence assembly stability and enzymatic activity maintenance [24, 26]. ASH2L also serves connection and regulatory functions: DPY30 primarily functions by binding to the SDI region at the C‐terminus of ASH2L, making ASH2L a critical node for DPY30 integration into the core complex and for regulating the distribution of H3K4me2/3 in vivo [31]. Cryo‐EM structural studies further reveal that the stable positioning of the KMT2A core complex on nucleosomes relies mainly on the anchoring role of RBBP5 and ASH2L. Both make extensive contacts with nucleosomal DNA and histone surfaces, thereby stabilizing the complex's binding to the nucleosome surface and promoting the H3K4 methylation reaction [24, 26, 32].

DPY30 is one of the WRAD core subunits of COMPASS‐like complexes, consisting of 99 amino acids in both humans and mice. DPY30 is the only subunit among the four core ones that lacks a direct connection to the KMT2 catalytic subunit. Instead, it is integrated into the complex through direct interaction with the SDI region at the C‐terminus of ASH2L. Structural studies show that the highly conserved C‐terminal domain of DPY30 mediates homodimerization and forms a hydrophobic groove that accommodates the amphipathic α‐helix of the ASH2L–SDI, stabilizing this interface and promoting complex assembly [24, 26, 32]. Despite its small size, DPY30 promotes complex assembly and catalytic output through its integration interface with ASH2L. Impairment of DPY30 in cells or knockout models is often accompanied by a significant decrease in H3K4me3 [24, 33, 34]. Mechanistically, the DPY30–ASH2L interaction not only affects local structure but can also promote the integration of ASH2L within the complex and restrict the dynamics of KMT2A on nucleosomes. Functionally, in vitro systems using free H3/peptides as substrates often show only a mild enhancement of KMT2 catalytic activity by DPY30, but its impact is differential at the gene level. Additionally, DPY30 is the only core subunit capable of forming homodimers and weak oligomers (primarily tetramers). The biological significance of this may be related to the regulation of complex conformation and stabilization on chromatin substrates, but the causal mechanisms in vivo require further elucidation [6, 35, 36].

The specific partner of the KMT2A/KMT2B complex, menin (MEN1), promotes cooperation between the KMT2A complex and LEDGF in the leukemia‐associated context, thereby stabilizing complex occupancy at target sites and maintaining aberrant transcriptional programs [37, 38, 39]. Mechanistically, LEDGF anchors to transcriptionally active chromatin through its PWWP domain recognizing active transcription‐related marks like H3K36me2/3, providing a crucial chromatin docking platform for menin –KMT2A [40].

KMT2C/D complex‐specific subunits include PTIP, PA1, NCOA6, and KDM6A/UTX, among others. These components collectively participate in complex recruitment, enhancer landscape establishment, and long‐range regulatory output, interacting with cell type‐specific transcription factor networks [41, 42]. UTX can form a cooperative network with KMT2D and the coactivator p300, promoting the acquisition of activation marks like H3K4me1 and H3K27ac at enhancers and enabling activation. This process can rely either on its H3K27 demethylase activity or proceed via nonenzymatic mechanisms in different contexts [43, 44]. KMT2F/G complexes primarily include the specific components CFP1 (CXXC1), WDR82, and HCF1. Among these, CFP1 uses its CXXC domain to selectively bind to unmethylated CpG islands (CGIs) and incorporates multiple histone modification signals to attach the complex to promoter regions. It is a key determinant in maintaining the genome‐wide H3K4me3 distribution pattern [6, 42, 45]. For instance, under nucleosomal substrate conditions, the PHD2 domain of CFP1 is crucial for the H3K4me2/3 reaction stimulated by H2B monoubiquitination (H2Bub) [27, 46, 47]. WDR82 recognizes the Ser5‐phosphorylated form of the RNA polymerase II CTD during transcription initiation, thereby promoting the recruitment of SET1A/B near transcription start sites (TSSs) and supporting promoter‐associated H3K4me3 deposition. Furthermore, multiple proteomic studies also categorize HCF1 as a related partner component, but its stability and functional contribution across different cell types and physiological or pathological conditions require further systematic analysis [24, 42, 48].

Impairment in assembly often leads to global or local decreases in H3K4 methylation and perturbs gene expression networks, thereby linking to developmental defects, neurodevelopmental abnormalities, and tumorigenesis. For example, ASH2L deletion in multiple models correlates with widespread reduction of promoter H3K4me3 and disruption of transcriptional programs, emphasizing the importance of complex stability for biological function output [49, 50].

#### 2.1.2 Global Architecture and Conserved Domains of KMT2

The C‐terminal catalytic module of KMT2 family members forms a conserved interaction network with the WRAD core subunit, thereby stabilizing the SET domain in a highly active conformation. Different KMT2 members harbor distinct combinations of domains in their N‐terminal to middle regions, which determine their interactions with DNA/nucleosomes, the transcription apparatus, and regulatory factors, thus shaping the targeting and functional specialization of each complex [6, 26, 34].

SAM is used by the SET catalytic domains of all KMT2 family members as the methyl donor for catalyzing the mono‐, di‐, and tri‐methylation of H3K4. Structural and biochemical studies consistently demonstrate that the isolated SET domain has limited activity. Its efficient catalysis relies on the assembly and allosteric activation by the WRAD core complex, and differences at the interfaces can lead to regulation of product specificity [22, 51]. Conserved flanking segments of the SET (often referred to as N‐SET and Post‐SET) help stabilize the catalytic module and participate in forming key regulatory interfaces. Therefore, they are often considered an important structural basis for the regulation of catalytic efficiency and product spectrum by core subunits such as WRAD/RBBP5–ASH2L [51, 52]. KMT2A–KMT2D typically carry noncatalytic domains such as AT‐hooks, CXXC zinc fingers, and multiple PHD domains, which are used for DNA/chromatin anchoring, histone modification reading, and PPIs. For example, PHD domains can participate in recognizing the H3K4 methylation state and enhance stable occupancy of the complex near promoters [6, 51]. For KMT2D, current clearer evidence indicates that its PHD6 can selectively recognize H4K16ac, linking KMT2D to the MOF–H4K16ac axis and enhancer function regulation [53, 54].

KMT2F/G are relatively shorter and lack most PHD, AT‐hook, or CXXC domains. However, their N‐terminal contain RNA‐related modules such as RRM. Cellular and in vitro evidence supports that KMT2F possesses RNA‐binding capability, and its N‐terminal can influence transcriptional regulation of CGI‐associated genes [9, 55]. Furthermore, nucleosomal H2B ubiquitination is widely recognized to enhance the catalytic efficiency of most KMT2 family complexes [51, 56]. Taspase‐1 can cleave some members, notably KMT2A/B, into N‐terminal and C‐terminal fragments that then form stable complexes using structural motifs like FYRN/FYRC, thereby influencing protein stability and functional assembly [38, 57]. Recent structural biology work (e.g., combining single‐particle cryo‐EM with cross‐linking mass spectrometry) has revealed the dynamic assembly mechanisms of KMT2 complexes on chromatin substrates. When the core complex binds to nucleosomes, it can adopt multiple occupancy and conformational states and respond to substrate contexts (such as H2Bub) and achieve catalytic activation through numerous protein–protein/protein–nucleosome contact points [24]. These studies provide a structural‐level completion of our understanding of KMT2 complexes, from subunit composition and conserved structures to their functional dynamic assembly process.

### 2.2 Physiological Functions of KMT2 Family

#### 2.2.1 KMT2 Activity and DNA Methylation

The KMT2 family constitutes the major H3K4 methyltransferases in mammals. As catalytic cores of COMPASS or COMPASS‐like complexes, KMT2 enzymes deposit H3K4me1/2/3 at promoters and enhancers, thereby maintaining open chromatin states and promoting transcriptional activation [5]. This family plays essential roles in gene regulation, cellular differentiation, and organismal development.

H3K4me3 is typically enriched near TSSs and correlates with promoter activity. H3K4me3 and DNA methylation exhibit a distinct mutually exclusive distribution at promoters, particularly at CGIs, which is intricately linked to histone‐tail reading mechanisms in the DNMT3 de novo methylation system [58, 59]. Mechanistically, the ADD domains of DNMT3A/3B and the cofactor DNMT3L preferentially bind unmethylated H3K4 (H3K4me0) and relieve autoinhibition, whereas H3K4me3 blocks ADD–H3 tail engagement and thereby suppresses catalytic activation of the DNMT3 complex at these loci. Thus, H3K4me3 not only marks transcriptional activity but can also functionally restrain the propensity for de novo DNA methylation at CGI promoters [58, 59, 60, 61].

**FIGURE 2:**
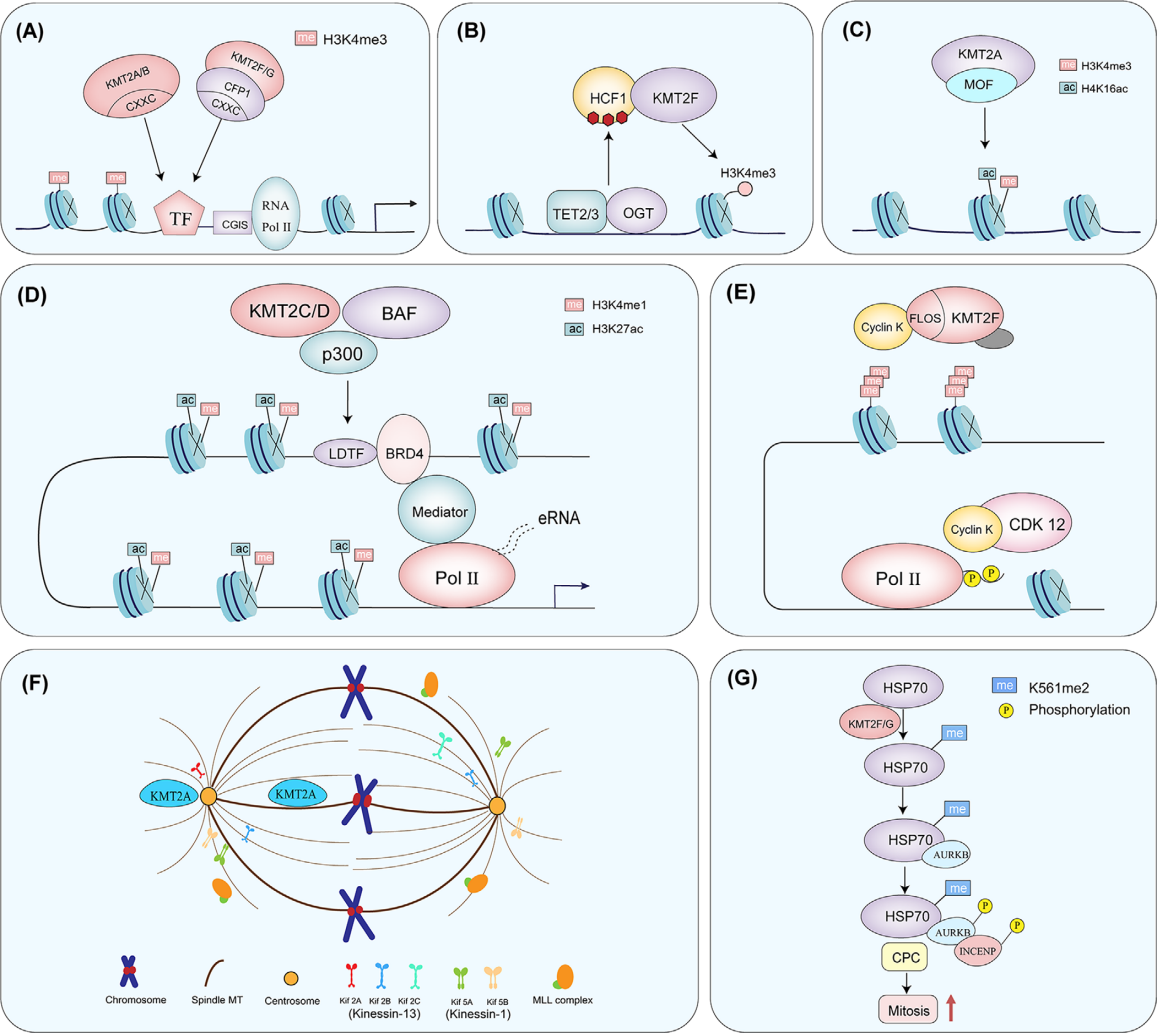
Functions of the KMT2 family. (A) KMT2 complexes promote the precise localization of H3K4me3 at CpG islands through CXXC zinc finger domains. (B) Interaction between TET2/3 and OGT drives O‐GlcNAcylation of proteins such as HCF1, thereby promoting KMT2 complex assembly and transcriptional activation. (C) KMT2A cooperates with MOF to regulate target gene expression. (D) Lineage‐determining transcription factors recruit KMT2C/D to initiate enhancer activation, followed by CBP/p300 and BRD4‐mediated assembly of the transcriptional machinery and transcriptional activation. (E) KMT2F maintains transcription of DNA damage response genes and genome stability under replication stress. (F) KMT2A participates in chromosome alignment, segregation, and spindle assembly. (G) KMT2F regulates cell cycle progression through dimethylation of lysine 561 on HSP70.

In addition to indirectly antagonizing DNMT3 activity, KMT2 complexes promote precise targeting of CGI‐associated H3K4me3 through CGI recognition mechanisms: KMT2A/B proteins utilize intrinsic CXXC domains to bind unmethylated CpG DNA directly, while KMT2F/G complexes rely on the CXXC domain of their subunit CFP1 to anchor the COMPASS complex to unmethylated CGIs [62] (Figure 2A). These distinct yet functionally convergent mechanisms ensure the establishment and maintenance of CGI‐associated H3K4me3 patterns [63]. Crucially, during early development, this dual‐targeting system supports a stable epigenetic state at key promoter CGIs, characterized by sustained transcriptional activity and high H3K4me3 in the presence of low DNA methylation. This configuration reinforces promoter resistance to silencing and stabilizes essential gene expression programs, thereby facilitating proper developmental progression [63, 64].

The TET2/3 proteins interact with OGT to drive the O‐GlcNAcylation modification of proteins such as HCF1. This alteration is crucial for the formation of the KMT2 complex. The TET proteins collaborate with OGT to facilitate the binding of KMT2F to chromatin, which subsequently mediates H3K4 trimethylation and transcriptional activation [65] (Figure 2B).

Overall, KMT2‐mediated H3K4 methylation and DNA methylation cooperate to shape CGI promoter epigenetic homeostasis through spatial mutual exclusion, targeting mechanisms, and functional coupling to DNA demethylation pathways, with important implications for gene expression and cell fate maintenance.

#### 2.2.2 Roles of KMT2 in Transcriptional Regulation

KMT2 family members promote gene transcription by depositing H3K4 methylation. KMT2A/B and KMT2F/G are preferentially enriched at promoters and catalyze H3K4me2/3, facilitating recruitment of RNA polymerase II, stabilization of transcription initiation complexes, and activation of highly expressed genes [66, 67].

Beyond its SET domain‐mediated H3K4 methylation, KMT2A can form a complex with the histone acetyltransferase MOF, promoting H4K16 acetylation at target loci—a modification strongly associated with open chromatin and robust transcriptional output [5] (Figure 2C). KMT2B‐mediated H3K4me3 acts independently of transcription; it promotes a relaxed chromatin state conducive to totipotency rather than directly influencing transcription [68].

KMT2C/D are major writers of enhancer‐associated H3K4me1/H3K4me2 in mammalian cells, representing a key epigenetic mark of enhancer priming. During differentiation and signal responses, enhancer activation typically proceeds through a staged epigenetic progression in which KMT2C/D play central roles. First, lineage‐specific or signal‐dependent transcription factors recognize prospective enhancer elements and help recruit COMPASS‐like complexes containing KMT2C/D to these chromatin regions. At this stage, KMT2C/D primarily deposit H3K4me1 or H3K4me2 on enhancer nucleosomes, establishing a “primed” state that provides an epigenetic foundation for subsequent activation. Upon differentiation cues or external stimuli, H3K4me1‐marked enhancers further recruit histone acetyltransferases CBP/p300, which install H3K27ac and drive the transition to a functionally active enhancer state. Activated enhancers become enriched for mediator, bromodomain‐containing protein 4 (BRD4), and RNA polymerase II and initiate transcription of enhancer RNAs (eRNAs) [6]. Meanwhile, through cooperation between KMT2C/D and multiple coactivators, stable chromatin looping forms between enhancers and their target promoters, facilitating effective loading of RNA polymerase II at promoters and productive transcriptional elongation [66, 69] (Figure 2D). Importantly, KMT2C/D contribute not only via H3K4 monomethyltransferase activity but also through structural coactivator functions that are critical for enhancer transcriptional competence and enhancer–promoter communication, highlighting their multifaceted roles in fine‐tuning enhancer‐driven gene regulation [66].

For KMT2F, beyond promoter‐associated H3K4me3 functions, accumulating studies emphasize a noncatalytic axis of transcriptional regulation. The N‐terminal structural modules of KMT2F (including the RRM and the WDR82‐binding region) are implicated in transcriptional control. Under replication stress or related stress conditions, KMT2F can functionally couple with the cyclin K–CDK12 axis to influence transcriptional maintenance of DNA damage response genes. This process aligns more closely with regulation of transcriptional processes and stress adaptation and should not be simplistically attributed to promoter H3K4me3 deposition alone [55, 70, 71] (Figure 2E).

#### 2.2.3 Regulation of KMT2 in the Cell Cycle and Development

In addition to their canonical roles in transcriptional activation, KMT2 family members can directly participate in cell‐cycle events such as replication checkpoints and mitosis, thereby influencing genome stability. For example, under DNA replication stress, ATR phosphorylates KMT2A at Ser516 during S phase. This modification weakens its interaction with SCF^Skp2^ and suppresses degradation, resulting in stabilization of KMT2A and increased chromatin association. Stabilized KMT2A has been linked to H3K4 methylation at late replication origin‐associated regions and suppression of CDC45 loading, thereby slowing replication and supporting execution of the mammalian S‐phase checkpoint [5, 72].

In addition to its involvement in DNA replication checkpoint regulation, the KMT2 complex also has mitosis functions that do not depend on transcription. For example, the KMT2A protein can localize to the mitotic spindle. By interacting with the microtubule depolymerizing motor protein KIF2A, it regulates the localization of KIF2A on the spindle. This further affects proper chromosome alignment and the spindle assembly process [51, 73, 74] (Figure 2F). KMT2F modifies the HSP70 protein by dimethylating it at the K561 site, which helps in controlling cell cycle progression. The dimethylated HSP70 binds to AURKB, which then connects with INCENP, activating AURKB kinase and forming a complex [51] (Figure 2G).

Evidence suggests that KMT2C can be drawn to DNA damage sites, where it facilitates local H3K4 methylation, promoting chromatin relaxation, enhancing DDR signaling, and attracting downstream DDR factors. In addition, alterations in KMT2C/D have been associated with tumor responses to PARP inhibitors, suggesting functional impacts on DNA repair capacity [75]. Furthermore, recent work shows that during cell fate transitions or differentiation, KMT2C/KMT2D‐dependent H3K4me1 not only marks enhancers but can also help shape replication programs: loss or catalytic impairment reduces replication timing changes and diminishes local replication origin activity, while exerting relatively limited effects on overall transcription. These findings suggest that H3K4me1 may link epigenetic state to replication dynamics in a manner partially independent of transcription [66].

In development, KMT2F is essential for epiblast‐stage progression and gastrulation in mouse embryos; loss of KMT2F leads to early postimplantation arrest, indicating an indispensable requirement for the KMT2 family in early developmental programs [68, 76].

Collectively, the KMT2 family forms an integrated regulatory network that spans transcription, replication, and cell‐cycle control—by modulating H3K4 methylation, influencing replication initiation, participating in checkpoint signaling, and affecting chromosome behavior through protein interactions. This network is critical for development and for maintenance of genome integrity.

## 3 Associations Between the KMT2 Family and Human Diseases

An increasing number of studies have demonstrated that KMT2 family members play a critical role in the pathogenesis and progression of various hematologic malignancies. Their aberrant alterations drive the development of leukemia and lymphoma by disrupting epigenetic homeostasis and transcriptional regulatory networks. In acute myeloid leukemia (AML), KMT2A rearrangements (KMT2A‐rearranged, KMT2A‐r) and certain tandem repeats are established molecular pathogenic events. In acute lymphoblastic leukemia (ALL), KMT2A‐r constitute a high‐risk molecular subtype in infant ALL. In diffuse large B‐cell lymphoma (DLBCL), frequent KMT2D inactivation mutations contribute to tumorigenesis by impairing enhancer function. These findings suggest that the KMT2 family exhibits specific pathogenic mechanisms in different hematologic tumor subtypes, providing important theoretical and translational foundations for precise molecular subtyping and targeted therapy of related diseases.

### 3.1 Nonsolid Tumors (Hematologic Malignancies)

#### 3.1.1 Acute Myeloid Leukemia

In recent years, large‐scale cohort‐targeted sequencing combined with functional studies have revealed that genetic alterations in KMT2 family members promote the development of AML by disrupting chromatin states and transcriptional programs. Among them, KMT2A‐r has the most robust evidence in genetics, mechanisms, and clinical translation [12, 77]. However, the alterations in KMT2C, KMT2D, and KMT2F members demonstrate subtype‐specific effects [78, 79].

KMT2A is located at chromosome 11q23 and exhibits structural fragility, making it prone to DNA breaks and chromosomal rearrangements. In acute leukemia, translocation breakpoints frequently cluster within specific breakpoint cluster regions (BCRs) in this area. This characteristic positions KMT2A as a critical driver in the pathogenesis of hematologic malignancies [80, 81, 82]. For AML and myelodysplastic syndromes, it has been confirmed through next‐generation sequencing (NGS) or fluorescence in situ hybridization detection that common alterations in KMT2A mainly include classic chromosomal rearrangements (translocations), partial tandem duplications (KMT2A‐PTD) [83, 84, 85], and copy number variations (CNVs) [39, 86].

**FIGURE 3:**
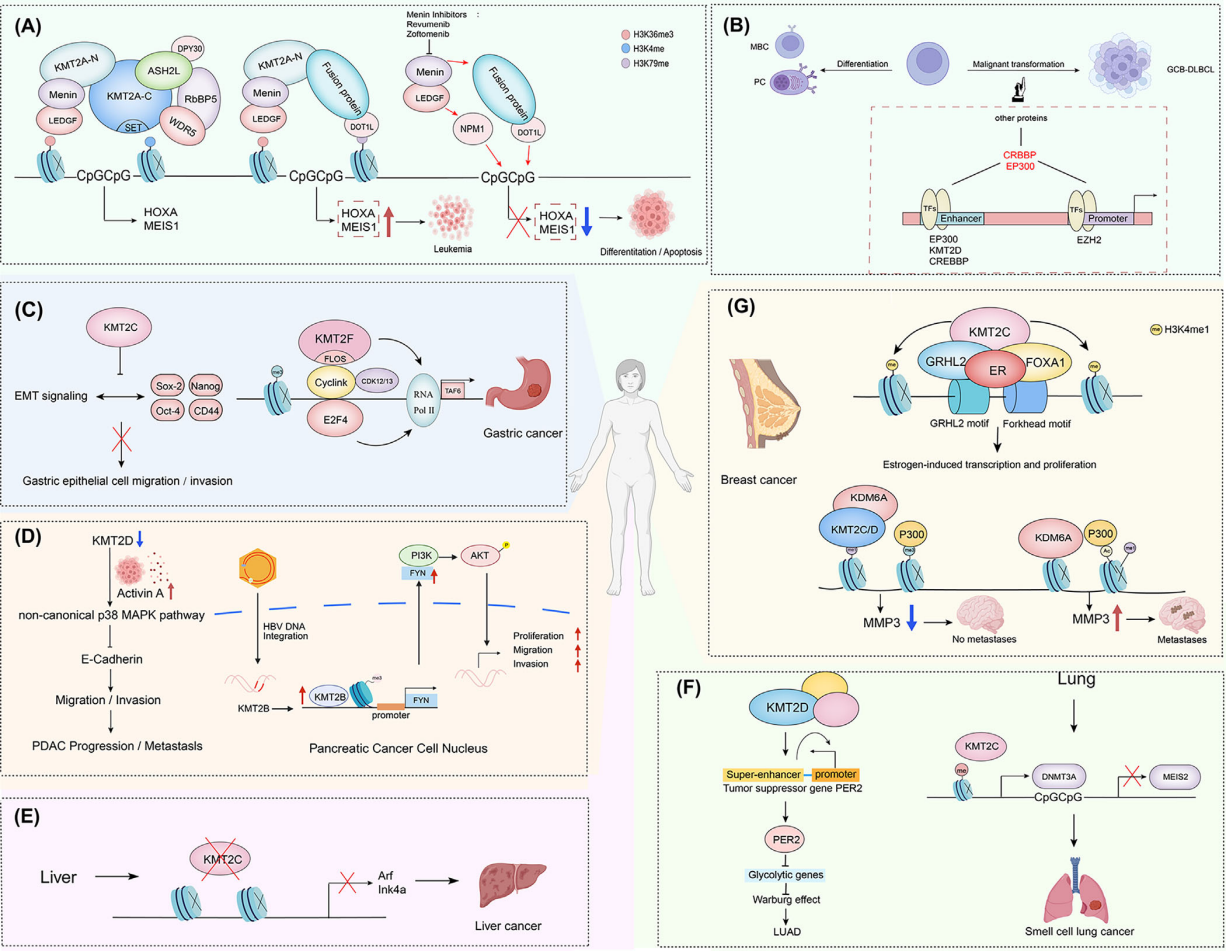
Association of the KMT2 family with diseases. (A) N‐terminal KMT2A fusion proteins aberrantly upregulate the expression of homeobox genes such as HOXA, MEIS1, thereby driving leukemogenesis. (B) Loss‐of‐function mutations in KMT2D may impair the maintenance of enhancer activity, thereby constraining the propensity of centroblasts to transform into DLBCL. (C) Reduced KMT2C expression enhances the migratory and invasive capacities of gastric epithelial cells by activating epithelial–mesenchymal transition signaling. KMT2F and its downstream effector TAF6 are positively regulated by the transcription factor E2F4, thereby promoting gastric cancer cell proliferation. (D) KMT2D and KMT2B promote pancreatic cancer progression through the activin A/p38–MAPK and FYN/PI3K/AKT pathways, respectively. (E) In hepatocellular carcinoma, KMT2C primarily drives tumorigenesis by inactivating key tumor suppressor genes such as Arf and Ink4a. (F) Loss of KMT2C promotes metastasis of small‐cell lung cancer by altering the cellular epigenetic state. KMT2D activates PER2 expression through super‐enhancers, thereby indirectly suppressing enhanced aerobic glycolysis in lung adenocarcinoma. (G) FOXA1 recruits KMT2C to specifically regulate epigenetic modifications at enhancer regions in breast cancer cells. Loss of KMT2C/D drives a metastatic phenotype by reshaping the enhancer epigenetic landscape at the MMP3 locus.

In AML, cases with KMT2A‐r constitutes a distinct aggressive subtype, typically characterized by poor prognosis, drug resistance, and high recurrence rates [39, 87]. KMT2A fusion proteins typically retain the KMT2A N‐terminus and targeting‐related domains (e.g., AT‐hooks and the CXXC domain) but lack the C‐terminal SET catalytic domain. Therefore, its pathogenicity does not depend on intrinsic histone modification activity, but rather drives a sustained oncogenic transcriptional program through aberrant recruitment of transcription coactivators and elongation complexes [88]. The core feature of this program is the abnormally high expression of HOX gene clusters (e.g., HOXA9) and their cofactor MEIS1 in myeloid stem/progenitor cells, leading to differentiation arrest and maintenance of leukemia dryness [14, 89] (Figure 3A).

In KMT2A‐r AML and NPM1‐mutant AML (hereafter abbreviated as NPM1‐m AML), the interaction between menin protein and the N‐terminal region of the KMT2A fusion protein is critical for maintaining this aberrant program, as it collaborates with chromatin factors (such as LEDGF/PSIP1) to stabilize KMT2A occupancy in the target gene region [90, 91, 92, 93]. And WDR5 binds to KMT2A and other KMT2 family members through its WIN binding pocket, stabilizing the assembly of the H3K4 methyltransferase complex at target gene loci [94]. In summary, the menin–KMT2A interaction primarily drives the aberrant transcriptional program, while the WDR5–KMT2A interaction maintains its epigenetic state, and together, they constitute key therapeutic dependencies in KMT2‐driven leukemia that are amenable to drug intervention.

#### 3.1.2 Acute Lymphoblastic Leukemia

Arising from B‐ or T‐lymphoid precursor cells, ALL is a highly varied blood malignancy and is among the most common leukemias found in children. Unlike AML, which is often characterized by disruption of transcriptional and epigenetic homeostasis, the molecular pathogenic events in ALL more frequently center on abnormalities of developmental stage‐specific transcription factors, key signaling pathways, and regulatory axes linked to treatment response [95]. In this setting, KMT2 family alterations are not universal drivers in ALL, but constitute indispensable pathogenic lesions in specific molecular subtypes. Among these, KMT2A‐r ALL has been studied most extensively and is highly enriched in infant ALL [96].

KMT2A‐r ALL is driven by fusion genes formed between KMT2A and diverse partner genes; in infants, common fusions include AFF1 (AF4 on chromosome 4), among others [96]. KMT2A fusion proteins depend on N‐terminal chromatin‐targeting capability to aberrantly assemble transcription activation and elongation‐related complexes and sustain leukemic transcriptional programs, while the menin–KMT2A interaction is an important basis for stable chromatin occupancy of these complexes and maintenance of leukemic transcriptional networks such as HOX/MEIS1 [96, 97]. In addition, in vitro and model studies indicate that KMT2A‐r ALL can undergo adaptive reprogramming under epigenetic therapeutic pressures such as DOT1L inhibition and acquire resistance, highlighting the plasticity and complexity of its transcriptional dependency and therapeutic escape mechanisms [97].

KMT2A‐r ALL exhibits limited efficacy and significant toxicity with conventional chemotherapy, prompting a shift in clinical strategy toward immunotherapy (such as blinatumomab) and small‐molecule inhibitors targeting the menin–KMT2A axis (such as revumenib). Early clinical studies have demonstrated both response activity and manageable safety profiles [12, 14, 98].

#### 3.1.3 Diffuse Large B‐Cell Lymphoma

DLBCL is among the most common aggressive non‐Hodgkin lymphomas in adults and is characterized by marked molecular heterogeneity, with substantial variability in clinical behavior and treatment response. Recent genomic and functional studies indicate that, in addition to abnormalities in classic signaling pathways such as BCR/NF‐κB, dysregulation of chromatin and epigenetic control is a major driver of DLBCL pathogenesis and a determinant of tumor phenotypes [99, 100, 101].

Among KMT2 family members, KMT2D stands out as the most clearly implicated and mechanistically supported contributor to the biology of germinal center (GC)‐derived B‐cell lymphomas, such as DLBCL. KMT2D mutations are frequently truncating or inactivating and are often considered initiating events. At enhancers, KMT2D predominantly deposits H3K4me1/me2 to prime enhancer activation, thereby supporting transcriptional networks and immune signaling response programs required for GC reactions [100, 102]. Rather than causing a uniform global reduction in H3K4 methylation, KMT2D inactivation is more prominently associated with selective impairment of enhancer H3K4me1 networks and reduced downstream transcriptional plasticity. This disrupts GC B‐cell state transitions and responses to antigen stimulation and T‐cell help signals, thereby increasing susceptibility to malignant transformation. KMT2D is also tightly functionally coupled to enhancer coregulators such as CREBBP/EP300, which maintain enhancer acetylation (e.g., H3K27ac) and can cooperate with KMT2D at enhancers. These factors are frequently comutated in GC‐derived lymphomas, can cooperate to promote lymphomagenesis in models, and may be accompanied by more immunosuppressive microenvironmental features, suggesting that a broader breakdown of enhancer activation modules represents an important molecular basis of this class of DLBCL [100, 103] (Figure 3B). By contrast, other KMT2 members such as KMT2C typically show lower mutation frequencies in DLBCL, and current evidence for their functional consequences and direct pathogenic roles is generally less substantial than that for KMT2D, warranting further systematic validation.

### 3.2 Solid Tumors

Members of the KMT2 family are key players in the evolution and advancement of various solid tumors. Frequent mutations in this family of genes across multiple cancer types indicate their extensive involvement and regulation of core tumor‐associated signaling pathways. As critical histone H3K4 methyltransferases, KMT2 family members profoundly influence tumor cell proliferation, differentiation, invasion, metastasis, and microenvironment remodeling by modulating chromatin states and gene expression. From hepatocellular carcinoma and gastric cancer in the digestive system to lung cancer in the respiratory system, and bladder cancer, prostate cancer, and breast cancer in the urogenital system, KMT2 family members exhibit functional heterogeneity—serving both as oncogenic drivers of malignant progression and as tumor suppressive factors maintaining genomic stability and cellular homeostasis. Their functional status is closely associated with tumor molecular characteristics, immune microenvironment, metabolic reprogramming, and therapeutic sensitivity, highlighting their central role in the tumor epigenetic regulatory network. These members represent promising therapeutic targets and prognostic biomarkers.

#### 3.2.1 Digestive System Diseases

Mutations and functional dysregulation of KMT2 family genes play key driving roles in multiple digestive‐system solid tumors. In gastric cancer, the overall mutation rate of KMT2 family genes is approximately 10.6% and is dominated by truncating loss‐of‐function mutations. KMT2‐mutant gastric cancers are characterized by elevated tumor mutational burden, microsatellite instability, and PD‐L1 expression. Their mutational spectra tend to co‐occur with genes involved in epigenetic regulation, DNA damage repair, and PI3K/MAPK pathways, yet show mutual exclusivity with TP53 mutations [11]. These features collectively shape an immune‐activated microenvironment, characterized by upregulated interferon–response pathways and increased immune‐cell infiltration. Studies have shown that in vitro knockdown of KMT2C promotes migration and invasion of gastric epithelial cells by promoting the EMT signaling pathway [104]. Notably, in gastric cancer, KMT2F can promote tumor cell proliferation independent of its canonical H3K4 methyltransferase catalytic activity: through its noncatalytic FLOS domain, KMT2F cooperates with E2F4 to upregulate TAF6 expression, thereby driving G1/S cell‐cycle progression and enhancing proliferation [55, 104] (Figure 3C). In KMT2‐mutant colorectal cancer, concurrent mutations within Wnt signaling, ERBB2/4, TGF‐β superfamily, and PI3K pathways suggest cooperative oncogenic potential of these pathways in specific epigenetic contexts [105].

In PDAC, beyond classic driver events such as KRAS and TP53, enhancer/promoter chromatin imbalance mediated by KMT2 complexes is considered an important epigenetic event that promotes invasion and metastasis [106, 107, 108]. In PDAC, KMT2D more commonly exhibits tumor‐suppressive features: downregulation or loss of KMT2D can induce Activin A secretion and activate noncanonical p38–MAPK signaling, thereby promoting cellular plasticity and invasive/metastatic behavior. This process is accompanied by EMT‐associated transcriptional reprogramming, often manifested as E‐cadherin downregulation and accelerated tumor progression [106]. Another study found that hepatitis B virus integration promotes the occurrence and development of pancreatic cancer by activating KMT2B expression, enhancing H3K4me3 modification in the FYN promoter region, and activating the PI3K/AKT pathway [109] (Figure 3D).

In hepatocellular carcinomas, KMT2C is mutated in more than 50% of cases, with these mutations also appearing in liver metastases. These mutations might improve the ability of hepatocellular carcinoma cells to home and colonize in the liver, suggesting that common KMT2C mutations/rearrangements not only drive primary hepatocellular carcinoma but may also contribute to the development of liver metastatic lesions [110] (Figure 3E).

#### 3.2.2 Respiratory System Diseases

In respiratory cancers, particularly lung cancer, members of the KMT2 family exhibit functional heterogeneity, and their aberrant expression is closely associated with tumor malignant progression and patient prognosis. Epigenetic modifiers frequently undergo loss‐of‐function mutations in lung cancer, yet their tumor‐suppressive functions are seldom clearly delineated. In lung cancer, the histone methyltransferase KMT2D is among the most frequently inactivated epigenetic modifiers.

Studies have shown that KMT2D can activate the expression of Per2 via super‐enhancers, thereby indirectly suppressing glycolysis‐related genes and ultimately limiting the enhancement of aerobic glycolysis (i.e., the Warburg effect) in lung adenocarcinoma (LUAD)—a key metabolic characteristic of cancer cells. The concurrent loss of KMT2D results in elevated expression of glycolytic genes, further highlighting its role as a suppressor in lung tumors. In terms of translational mechanism, mouse xenograft models have confirmed that the glycolysis inhibitor 2‐DG can significantly inhibit the tumorigenic capacity of LUAD cell lines carrying KMT2D‐inactivating mutations. This indicates that tumors with KMT2D deficiency are therapeutically vulnerable to glycolysis inhibitors (such as 2‐DG), providing a potential strategy for targeting metabolic pathways [111] (Figure 3F).

Lung squamous cell carcinoma (LUSC), a key subtype of lung cancer, has been found to involve KMT2D as a significant epigenetic regulator in its tumorigenesis. KMT2D loss can lead to the transformation of lung basal cell organoids into LUSC, with its deficiency boosting RTK, EGFR, and ERBB2 activation by modifying chromatin structure to lower protein tyrosine phosphatase expression. These changes collectively lead to a significant elevation in oncogenic RTK–RAS signaling [112]. Besides KMT2D, KMT2C is another frequently mutated epigenetic regulatory gene in lung cancer. In LUAD, low expression of the KMT2C gene is significantly associated with shortened overall survival (OS) in patients [113]. Additionally, this gene's mutation rate is considerably elevated in smokers as opposed to nonsmokers and correlates with younger LUAD patients. Additionally, KMT2C is a key mutated gene shared between primary lung cancer lesions and metastases in multiple organs (such as the brain, bone, and liver), suggesting its potential role in driving the metastatic process [114].

#### 3.2.3 Genitourinary System Diseases

In multiple genomic cohorts of bladder urothelial carcinoma, KMT2C and KMT2D are the most frequently mutated genes. Their alterations are predominantly inactivating mutations, including truncations, frameshifts, and splice‐site variants, showing consistency across sequencing cohorts [115, 116, 117]. Such alterations often co‐occur with enhancer‐associated epigenetic regulators such as KDM6A(UTX), suggesting a systematic impairment of an enhancer‐centered chromatin regulatory module in bladder cancer [118]. Mechanistically, KMT2C/KMT2D primarily contribute to establishment of enhancer‐associated H3K4me1, while KDM6A catalyzes H3K27me3 demethylation. These changes are consistent with dysregulated expression of urothelial differentiation‐associated genes, supporting a close link to enhancer‐level chromatin dysregulation [119, 120]. Functional studies and genetic models further indicate that loss of KMT2C or KMT2D in urothelium reduces the stability of urothelial differentiation states and significantly increases susceptibility to carcinogenic stimuli [119].

In prostate cancer, epigenetic dysregulation is increasingly recognized as a molecular background that promotes lineage plasticity and therapy resistance, with KMT2C alterations standing out as particularly prominent. Available evidence indicates that loss or inactivating mutations of KMT2C can promote transition toward androgen receptor (AR)‐independent states under selective pressure from androgen deprivation or AR pathway inhibition and are associated with acquired resistance to AR‐targeted therapies [121, 122]. Mechanistically, this phenotypic switch is linked to reprogramming of enhancer and transcriptional regulatory networks, weakening lineage‐maintenance programs while activating alternative transcriptional states [123, 124]. Integrative analyses using in vivo genetic models and clinical cohorts further suggest that KMT2C alterations are associated with increased aggressiveness, higher metastatic risk, and poor prognosis [125]. Thus, current evidence supports a key and context‐dependent tumor‐suppressive role for KMT2C in limiting lineage remodeling by maintaining enhancer–transcriptional homeostasis during prostate cancer progression and treatment resistance.

The KMT2C gene exhibits a high mutation frequency in breast cancer, and these mutations may contribute to tumorigenesis and progression by influencing chromatin function and transcriptional mechanisms. Studies indicate that KMT2C mutations are particularly common in HR+/HER2− breast cancer and are essential for the regulation of the estrogen receptor ERα, potentially driving hormone‐dependent cancer cell proliferation [126]. KMT2C collaborates with FOXA1 to monomethylate H3K4 at enhancers, activating transcription. Furthermore, KMT2C and KMT2D can interact with the estrogen receptor ER to coregulate the expression of downstream target genes when estrogen E2 is present, thereby promoting breast cancer progression [127]. Additionally, studies have shown that chromatin remodeling and histone modification alterations resulting from KMT2C/D mutations indirectly affect MMP3 expression through KDM6A, thereby inducing the molecular mechanism underlying distant metastasis in triple‐negative breast cancer. Meanwhile, inhibiting the activity of KDM6A can reduce MMP3 expression and may prevent brain metastasis caused by KMT2C/D mutations [128] (Figure 3G).

Ovarian cancer exhibits high molecular and histologic heterogeneity, in which KMT2 family functions show clear subtype dependence [129, 130]. Molecular subtyping and functional studies indicate that KMT2C and KMT2D abnormalities can contribute to remodeling of chromatin and transcriptional programs in certain ovarian cancer subtypes and associate with tumorigenesis and progression, although the impact is not universal across all subtypes [131, 132]. Mechanistic work further suggests that KMT2F can cooperate with the Notch signaling pathway to enhance Notch‐dependent transcriptional activity, thereby promoting ovarian cancer cell proliferation and tumor progression [133]. In addition, some studies have proposed that KMT2A may participate in establishing malignant phenotypes by regulating EMT‐related programs, although these effects remain constrained to specific models and molecular backgrounds. Overall, the central impact of the KMT2 family in ovarian cancer may lie in remodeling promoter‐ and enhancer‐level transcriptional programs, shaping tumor phenotypes and progression in a subtype‐specific manner [134].

### 3.3 Neurologic and NDDs

Unlike the acquired somatic mutations commonly observed in cancer, KMT2 family genes systematically influence critical developmental programs during the initial stages of individual development. These mutations disrupt epigenetic homeostasis and significantly impact the development, differentiation, and functional maintenance of the nervous system. Studying these relatively well‐characterized diseases caused by single‐gene mutations provides a unique and clear perspective for elucidating the precise regulatory functions of the KMT2 family in embryogenesis and neural development, thereby further revealing the molecular roles of this family under physiological conditions.

#### 3.3.1 KMT2B‐Related Dystonia

Heterozygous pathogenic variants in KMT2B can cause an inherited movement disorder with dystonia as the core phenotype, termed KMT2B‐related dystonia (DYT‐KMT2B). Reported pathogenic variants are predominantly loss‐of‐function, broadly supporting haploinsufficiency as the principal disease mechanism. Common variant types include nonsense and frameshift variants, splice‐altering variants, and copy‐number deletions (CNVs/microdeletions) affecting part or all of the gene [135, 136].

Clinically, DYT‐KMT2B typically presents in childhood and shows progressive worsening, consistent with dose‐dependent regulatory impairment due to KMT2B loss‐of‐function. Cohort studies highlight that KMT2B‐associated dystonia leads to speech and articulation impairments in affected patients [137]. Notably, DYT‐KMT2B is commonly accompanied by neurodevelopmental and neuropsychiatric features, suggesting that KMT2B‐driven pathogenic mechanisms extend beyond motor circuitry to involve broader neurodevelopmental transcriptional program disturbances. Clinically, the disorder therefore often aligns with complex or syndromic dystonia [136].

For the treatment of DYT‐KMT2B, deep brain stimulation of the internal globus pallidus (GPi‐DBS) can significantly improve overall motor symptoms and functional outcomes. However, response magnitudes vary substantially across individuals, suggesting that phenotypic and molecular backgrounds may influence treatment effects [137, 138]. Additionally, studies have demonstrated an association between DNA methylation aberrations and the age at onset of KMT2B‐deficient dystonia, providing a functional adjunct for molecular diagnosis [139].

#### 3.3.2 KS (KS Type 1)

KS is a multisystem disorder with neurodevelopmental abnormalities as a central feature. Typical manifestations include characteristic facial features, developmental delay or intellectual disability, skeletal anomalies, persistent fetal fingertip pads, and involvement of organs such as the heart and kidneys; clinical diagnosis is usually supported by molecular genetic testing [140].

Among established genetic causes, KS is primarily due to pathogenic variants in KMT2D (KS1, autosomal dominant) or KDM6A (KS2, X‐linked). Most cases are due to changes in KMT2D, whereas KDM6A‐associated cases show more pronounced phenotypic heterogeneity with respect to sex and variant type [141, 142, 143]. From the perspective of the KMT2 family, KS1 represents a prototypical epigenetic disorder driven by KMT2D loss‐of‐function or haploinsufficiency. KMT2D maintains enhancer‐level H3K4 methylation states and cooperates with KDM6A‐mediated H3K27 demethylation to regulate transcriptional activation programs [144, 145].

In clinical practice, diagnosing or evaluating diseases associated with KMT2D/KDM6A relies critically on detecting sequence variants or copy number variants in these two genes. Current management remains largely symptomatic and focused on systematic surveillance of complications, while disease‐modifying interventions targeting epigenetic dysregulation remain at preclinical or early exploratory stages [146, 147].

#### 3.3.3 Wiedemann–Steiner Syndrome

WSS (or WDSTS) represents a multisystem chromatin related disorder. Its primary clinical feature is neurodevelopmental impairment, with a genetic basis that clearly implicates KMT2A. Current evidence consistently indicates that WSS is mainly caused by heterozygous pathogenic variants in KMT2A, with haploinsufficiency due to loss‐of‐function as the core mechanism. This disrupts KMT2A‐mediated H3K4 methylation‐associated transcriptional regulation and affects neurodevelopment in a dose‐dependent manner [148, 149]. At the molecular level, KMT2A forms functional coupling with demethylases such as KDM5C in transcriptional regulation; disruption of this balance can perturb neuronal gene expression and synapse‐related programs, thereby contributing to the neurodevelopmental phenotypes of WSS [150]. Currently, the definitive diagnosis of WSS relies on genetic testing, and its treatment is primarily symptomatic [151].

## 4 Diagnostic and Therapeutic Implications of the KMT2 Family

The growing understanding of KMT2 family dysregulation in human disease has paved the way for its clinical application in diagnosis, prognosis, and targeted intervention. This section first evaluates the diagnostic and prognostic value of KMT2 family alterations across different diseases, and then systematically reviews current therapeutic strategies targeting KMT2‐associated pathogenic mechanisms, including PPI inhibitors, targeted protein degraders (TPDs), and transcriptional program inhibitors.

### 4.1 Diagnostic and Prognostic Value of KMT2 Family Alterations

Members of the KMT2 family play a key role in regulating gene expression and maintaining chromatin accessibility [5, 152]. Genetic alterations of KMT2 family members—particularly mutations and rearrangements involving KMT2A, KMT2C, and KMT2D—are closely associated with the development and progression of a wide range of malignant tumors and congenital hereditary disorders, conferring important clinical significance in molecular diagnosis and prognostic assessment [152, 153, 154].

KMT2 family members have demonstrated significant diagnostic and prognostic value across various diseases [155]. KMT2A‐r are well‐established diagnostic and classification markers in both pediatric and adult acute leukemia [39, 88]. In solid tumors, frequent mutations in KMT2C and KMT2D are closely associated with tumor initiation, progression, and patient outcomes [119, 156]. Specifically, KMT2D plays a critical role in regulating enhancer‐associated H3K4 monomethylation and modulates the expression of tumor suppressor genes such as PTEN and p53, thereby restraining tumor proliferation and metastasis. This positions KMT2D as a promising diagnostic marker and potential therapeutic target in cancers like bladder carcinoma [157]. Beyond oncology, germline alterations in KMT2D and KMT2A are causative for KS and WSS, respectively, underscoring their importance in developmental disorders and further supporting their utility in clinical diagnosis [145, 158].

With respect to prognosis and prediction of therapeutic response, multiple retrospective and pan‐cancer analyses have shown that patients harboring KMT2 mutations who do not receive immune checkpoint inhibitors (ICIs) tend to exhibit poorer OS, although the independent prognostic value of KMT2 mutations displays substantial heterogeneity across different cancer types. Notably, among patients treated with ICIs, objective response rates (ORR) and durable clinical benefit are significantly improved in those with KMT2 mutations [159, 160]. KMT2 family member alterations have been noted as indicators of a favorable response to ICI treatment [160, 161, 162]. Tumor immune microenvironment analyses have revealed that tumors harboring KMT2 mutations are characterized by higher levels of immune cell cytotoxicity and enhanced immune cell infiltration, exhibiting a typical “immune‐inflamed” or “hot tumor” phenotype [159].

Collectively, KMT2 family mutations function not only as molecular genetic markers but also reflect key features of the tumor microenvironment, thereby providing a basis for prognostic evaluation and personalized therapeutic strategies [155, 159]. Nevertheless, given the marked heterogeneity of prognostic implications associated with KMT2 alterations across different diseases—such as leukemia, colorectal cancer, and NDDs—their clinical application should be carefully interpreted in the context of specific disease types and treatment regimens. A comprehensive summary of the diagnostic and prognostic value of KMT2 family members across various human diseases is provided in Table 1.

**TABLE 1:** Diagnostic and prognostic value of KMT2 family in human diseases.

KMT2 member	Disease type/subtype	Types of mutations	Diagnostic/prognostic value	Clinical features	References
KMT2A	AML/ALL; WSS	Leukemia: KMT2A‐r, KMT2A‐PTD, CNVs; WSS: heterozygous loss‐of‐function variants including nonsense, frameshift, and splice‐site mutations	Leukemia: KMT2A‐r defines distinct WHO/ICC genetic subtypes of AML and ALL, used for diagnosis, risk stratification, and prognosis; fusion partner identity further refines prognostic prediction. WSS: caused by heterozygous pathogenic KMT2A variants	Leukemia: hyperleukocytosis, frequent CNS involvement. WSS: neurodevelopmental impairment, growth retardation, developmental delay, hypotonia, hypertrichosis cubiti, short stature, distinctive facial features, dental anomalies	[39, 99, 148, 149, 163, 164, 165, 166, 167, 168]
KMT2B	DYT‐KMT2B (DYT28); NDDs	Heterozygous loss‐of‐function variants (nonsense, frameshift, splice‐site), missense variants, and gene deletions	KMT2B is a key diagnostic gene for DYT28; molecular testing confirms diagnosis and informs prognosis and DBS therapy.	DYT‐KMT2B: progressive childhood‐onset dystonia, developmental delay, intellectual disability, microcephaly, short stature, speech and motor coordination deficits; NDDs: neuropsychiatric features	[135, 136, 137, 138, 169, 170, 171]
KMT2C	NDDs; multiple solid tumors	Somatic loss‐of‐function mutations, focal deletions; rare germline variants	Germline mutations confirm diagnosis of NDDs; somatic mutations may serve as prognostic marker in cancers.	Developmental delay, intellectual disability, hypotonia, craniofacial dysmorphism, autistic features; in cancer, tumor suppressor loss	[172, 173, 174, 175, 176]
KMT2D	Kabuki syndrome type 1 (KS1); NDDs; cancer	Germline: heterozygous loss‐of‐function variants (nonsense, frameshift, splice‐site), missense variants; small deletions or insertions	Key diagnostic gene for KS1; somatic mutations may have prognostic implications in tumors.	KS: intellectual disability, growth retardation, distinctive facial features, skeletal anomalies, cardiac defects, immune dysfunction Cancer: enhancer dysregulation and transcriptional instability	[140, 141, 142, 143, 144, 145, 146, 154, 177, 178, 179, 180]
KMT2F	Neurodevelopmental disorder with speech impairment and dysmorphic facies (NEDSID), epilepsy, schizophrenia	Heterozygous loss‐of‐function, missense variants	High‐risk pathogenic factors for schizophrenia and NDDs	Intellectual disability, developmental and speech delay, epilepsy, schizophrenia, and behavioral abnormalities	[48, 181, 182, 183, 184, 185]
KMT2G	Intellectual developmental disorder with seizures and language delay (IDDSELD); NDDs; autism spectrum disorder	Heterozygous loss‐of‐function variants, missense variants	Genetic causes of epilepsy with developmental delay syndrome	Global developmental delay, speech and language impairment, onset of seizures, accompanying behavioral abnormalities, facial dysmorphism, tapering fingers, pigmentary skin changes	[186, 187, 188, 189, 190]

### 4.2 Targeted Therapeutic Strategies for KMT2‐Associated Diseases

Based on a deep understanding of the pathogenic mechanisms of the KMT2 family in various diseases, translating this knowledge into clinical intervention targets has become a core task in translational medicine. The current therapeutic strategies targeting KMT2 dysregulation can be broadly categorized into three principal directions: (1) direct disruption of critical PPIs within KMT2 complexes (e.g., menin–KMT2A, WDR5–KMT2A); (2) targeted degradation of complex scaffolds or oncogenic fusion partners to induce profound epigenetic reprogramming; (3) inhibition of downstream transcriptional co‑activators or epigenetic “readers/writers” that sustain aberrant gene‑expression programs. The continuous development and refinement of these therapeutic strategies mark the transition of epigenetic dysregulation treatments from basic research to clinical translation. Detailed clinical trial information (including trial identifiers, efficacy data, and specific response rates) for mentioned inhibitors and combination regimens are systematically compiled in Table 2.

**TABLE 2:** Summary of clinical trials for targeted monotherapy and combination regimens in KMT2‐related diseases.

Inhibitor type	Drug name	Clinical trial identifier	Mechanism	Disease model	Effect	References
Menin inhibitors	Revumenib (SNDX‐5613)	NCT04065399 (Phase I/II)	Block the KMT2A–menin interaction downregulates MEIS1/HOX expression	R/R KMT2A‐r or NPM1‐m AML	(Phase I): 53% overall response rate, ORR was 59% in KMT2Ar and 36% in NPM1‐mutated patients (Phase II): 63.2% overall response rate, median CR/CRh duration was 6.4 months, with 68.2% of patients achieving MRD negativity.	[14, 191]
Menin inhibitors	Ziftomenib (KO‐539)	NCT04067336 (Phase I/II)	Inhibits KMT2A/MLL protein complex	Adult patients with R/R AML	CR/CRh rate was 25%, the CRc rate was 33% with 75% MRD‐ and ORR was 42% in participants treated with 600 mg ziftomenib.	[192, 193]
Menin inhibitors	Bleximenib (JNJ‐75276617)	NCT04811560 (Phase I)	Inhibits KMT2A–menin Protein–protein interaction	R/R acute leukemia harboring KMT2A or NPM1 alterations	ORRs of 40–50% at higher dose levels, frequent CRc responses, MRD negativity, and sustained clinical benefit.	[194]
Menin inhibitors	BMF‐219	NCT05153330 (Phase I)	Covalent menin inhibitor	R/R KMT2A‐r or NPM1‐m acute leukemia; DLBCL, MM, CLL/SLL	CR was achieved in two of five participants with evaluable efficacy.	[195]
Menin inhibitors	DSP‐5336	NCT04988555 (Phase I/II)	Inhibits menin–MLL protein interaction	Adult patients with R/R acute leukemia characterized by KMT2A‐r, NPM1‐m, and other HOXA9/MEIS1 driven leukemia subsets	Objective response rate was 59.1% and CR/CRh rate was 22.7% in 22 participants with KMT2A‐r CR with incomplete recovery.	[196]
Menin inhibitors	BN‐104	NCT06052813 (Phase I/II)	Target and disrupt the protein–protein interaction between menin and MLL	R/R KMT2A‐r AML	89% overall response rate with 33.3% CR/CRh rate in KMT2A‐r participants	[197]
DOT1L inhibitors	Pinometostat (EPZ‐5676)	NCT02141828 (Phase I)	DOT1L histone methyltransferase (HMT) inhibition via a SAM‐competitive mechanism	R/R leukemias bearing a rearrangement of the KMT2 gene	No objective responses despite sustained target H3K79 methylation inhibition	[198]
DOT1L inhibitors	Pinometostat (EPZ‐5676)	NCT01684150 (Phase I)	DOT1L histone methyltransferase (HMT) inhibition via a SAM‐competitive mechanism	Adult patients with R/R leukemias with KMT2A‐r	Rare CRs (4%) with low overall response rates, despite effective H3K79me2 suppression	[199]
LSD1 inhibitors	Iadademstat (ORY‐1001)	EudraCT No.: 2013‐002447‐29 (Phase I)	Potent covalent inhibitor of LSD1, which induces differentiation of AML cells and promises antitumor activity	R/R KMT2A‐r AML	Iadademstat exhibits a good safety profile together with signs of clinical and biologic activity.	[200]
Combination therapy	Revumenib + venetoclax	NCT06284486 (Phase I/II)	Menin–KMT2A interaction inhibition and BCL‐2 to suppress KMT2A‐dependent leukemogenic transcriptional programs and induce BCL‐2‐dependent apoptosis	KMT2A‐r, NPM1‐m, or NUP98‐r AML	No formal efficacy data reported yet	[201]
Combination therapy	Revumenib + ASTX727 + venetoclax	NCT05360160 (Phase I/II)	Menin–KMT2A interaction inhibition combined with BCL‐2 inhibition and DNA hypomethylation to enhance apoptosis and eradicate leukemic stem cell programs	R/R and unfit AML patients, enriched for KMT2A‐r/NPM1‐m/NUP98‐r	ORR was 88%, CR/CRh rate was 58%, MRD negativity was achieved in 74% of responders and 93% of patients with CR/CRh, respectively.	[202]
Combination therapy	Revumenib + venetoclax + azacitidine	NCT03013998 (Phase I)	Menin inhibition (revumenib) combined with BCL‐2 inhibition (venetoclax) and DNA hypomethylation (azacitidine)	NPM1‐m or KMT2A‐r AML	CRc rate was 81.4%, ORR was 88.4% and the rate of CR was 67.4%, MRD negativity in all evaluable responders.	[203]
Combination therapy	Revumenib + gilteritinib	NCT06222580 (Phase I)	Revumenib disrupts the menin–KMT2A interaction to inhibit HOX/MEIS1‑driven leukemogenesis; gilteritinib inhibits FLT3 signaling in FLT3‑mutated AML.	R/R FLT3‐mutated AML and concurrent KMT2A‐r or NPM1‐m	Preliminary responses: among evaluable patients, 1 MRD‐negative CR, 1 PR, 1 NR; no dose‐limiting toxicities (DLTs) observed	[204]
Combination therapy	Ziftomenib + venetoclax/azacitidine	NCT05735184 (Phase I)	Ziftomenib has demonstrated clinical activity as both monotherapy and in combination.	Adults with R/R NPM1‐m or KMT2A‐r AML	65% ORR (NPM1‑m) and 41% (KMT2A‑r), 48% CRc and 28%, respectively	[205, 206]
Combination therapy	Ziftomenib + venetoclax + azacitidine	NCT06397027 (Phase I)	Disruption of the menin–KMT2A oncogenic transcriptional complex by ziftomenib, combined with BCL‐2 inhibition (venetoclax) and hypomethylating epigenetic modulation (azacitidine)	Acute leukemias with KMT2A‐r, NPM1‐m, or NUP98‐r	No formal efficacy data reported yet	[207]
Combination therapy	Ziftomenib + fludarabine + Cytarabine	NCT06376162 (Phase I)	Menin–KMT2A interaction inhibitor in combination with chemotherapy (FLA regimen)	R/R KMT2A‐r, NUP98‐r, or NPM1‐m acute leukemia	No formal efficacy data reported yet	[208]
Combination therapy	Bleximenib + cytarabine + daunorubicin/idarubicin	NCT05453903 (Phase I)	Menin–KMT2A interaction inhibitor in combination with “7+3” IC regimen	Newly diagnosed NPM1‐m or KMT2A‐r AML	Preliminary antileukemic activity observed: high overall response rates (ORR: 93% and CR/CRh: 79–86% in small cohorts).	[209]
Combination therapy	Pinometostat + azacitidine	NCT03701295 (Phase I/II)	Pinometostat disrupts aberrant H3K79 methylation driven by KMT2A fusion proteins, combined with azacitidine to reverse dysregulated epigenetic marks and potentially enhance antileukemic activity.	R/R KMT2A‐r AML	No formal efficacy data reported yet.	[210]
Combination therapy	Lestaurtinib + chemotherapy	NCT00557193 (Phase III)	Inhibits FLT3 signaling overexpressed, enhancing chemotherapy‐induced apoptosis	KMT2A‐r infant ALL	No improvement in 3‐year EFS or OS compared with chemotherapy alone (3‐year EFS 36 vs. 39%; *p* = 0.67)	[211]
Combination therapy	Multiagent chemotherapy protocol	NCT00550992 (Phase III)	Cytotoxic chemotherapy targeting DNA synthesis and topoisomerase activity	KMT2A‐r infant ALL	No significant improvement with AML‐like intensification; 6‐year EFS 46.1%, OS 58.2%; ADE + MAE vs. IB DFS 39.3 vs. 36.8%	[212]
Combination therapy	Quizartinib + cytarabine + etoposide	NCT01411267 (Phase I)	FLT3 inhibition (quizartinib) combined with DNA synthesis inhibition and topoisomerase II blockade (cytarabine + etoposide)	R/R leukemia including KMT2A‑r ALL characterized by high FLT3 expression	CR 2/17, SD 10/17, PD 3/17; FLT3‐ITD patients 3/7 achieved CR/CRp/CRi; regimen tolerable with modest overall efficacy	[213]

#### 4.2.1 PPI Inhibitors

##### 4.2.1.1 Menin–KMT2A Interaction Inhibitors

Therapeutic strategies targeting the menin–KMT2A interaction have promoted the development of precision therapies directed at KMT2A‐associated transcriptional complexes and their downstream HOX/MEIS1 program [214, 215]. By binding with high affinity to the KMT2A binding pocket of menin, oral small‐molecule Menin inhibitors block the menin–KMT2A interaction, thereby inhibiting the proliferation of leukemia progenitor cells and inducing differentiation. These inhibitors demonstrate significant efficacy against leukemia subtypes dependent on HOXA/MEIS1 aberrant activation, such as those with KMT2A‐r and NPM1‐m [216, 217]. Currently, several menin inhibitors have entered clinical research stages, including revumenib (SNDX‐5613), ziftomenib (KO‐539), DSP‐5336, BMF‐219, and JNJ‐75276617 [218].

Revumenib is the initial menin inhibitor that has received approval [219]. The Phase I/II trial (NCT04065399) demonstrated significant responses and deep molecular remission in patients with relapsed/refractory leukemia with KMT2A‐r or NPM1‐m. Safety analysis indicated that revumenib was well tolerated, with adverse events including asymptomatic QTc prolongation and differentiation syndrome (DS), which could be managed with steroids and close monitoring. Additionally, the Phase I dose‐escalation study showed pharmacokinetic analyses demonstrated that revumenib exposure increased proportionally with dose [14, 191, 220]. These results suggest that revumenib demonstrates clinically meaningful efficacy in patients with heavily pretreated or relapsed/refractory disease.

Ziftomenib disrupts the menin–KMT2A interaction, interfering with oncogenic signaling and inducing differentiation in leukemia cells harboring KMT2A‑r or NPM1‑m [221, 222]. The Phase I/II KOMET‐001 (NCT04067336) clinical trial focuses on KMT2A‐r or NPM1‐m R/R AML. The Phase Ia dose‐escalation stage showed initial signs of clinical activity across dose levels, including marrow blast reduction and hematologic improvements. The Phase Ib determined that 600 mg/day is the recommended dose for Phase II, and therapeutic responses including complete remission were observed at this dose, with some patients achieving minimal residual disease negativity [193, 223, 224]. Although monotherapy demonstrates promising activity, primary or acquired resistance may still occur, and ongoing trials are exploring combination strategies with hypomethylating agents (HMAs), BCL‑2 inhibitors, and other targeted therapies.

##### 4.2.1.2 WDR5–KMT2A Interaction Inhibitors

WDR5 is a core scaffold subunit of the KMT2 complex, helping to assemble and stabilize the multiprotein complex that catalyzes H3K4 methylation [94, 225]. In KMT2A‐r leukemia, the fusion proteins rely on multiple epigenetic coregulators, including WDR5, to maintain active chromatin and aberrant transcription at oncogenic loci. Disrupting the WDR5–KMT2A interaction significantly downregulates the expression of key oncogenes such as HOXA9 and MEIS1, resulting in growth inhibition and induction of differentiation [225, 226, 227, 228].

Based on the minimal active peptide sequence of the WIN motif (ARA), a series of high‐affinity peptide mimetics have been developed, including MM‐101, MM‐102, MM‐103, and the cyclic peptide MM‐401 [229]. MM‐401 has been shown to bind WDR5 with low nanomolar affinity at the WIN site, effectively inhibiting histone H3K4 methyltransferase activity and downregulating oncogenic gene transcription, thereby suppressing proliferation and inducing cell cycle arrest in KMT2‐r leukemia cells [228, 230]. Furthermore, the cyclic peptide Ac7, optimized from the endogenous microprotein EMBOW structure, exhibits stronger binding affinity and protease stability, significantly inhibiting leukemia cell growth and demonstrating excellent antitumor activity and low toxicity in animal models [227]. In terms of small molecule inhibitors, a series of WDR5‐010 compounds, obtained via high‐throughput screening and structurally optimized to generate the representative chemical probe OICR‐9429, specifically bind to the WDR5 WIN pocket. This interaction disrupts WDR5–KMT2A binding and selectively suppresses proliferation of cancer cells, including those harboring TP53 gain‐of‐function mutations in preclinical models [231, 232, 233, 234].

Targeting the WDR5–KMT2A interaction by disrupting the oncogenic epigenetic maintenance mechanism complements the menin–KMT2A targeting strategy. Future efforts should focus on structural optimization and combination therapy strategies to drive its clinical translation.

#### 4.2.2 Targeted Protein Degraders

TPD, utilizing proteolysis‐targeting chimeras (PROTACs) and molecular glues, is a groundbreaking therapeutic approach. Through the recruitment of E3 ubiquitin ligases to initiate the ubiquitination and following proteasomal degradation of specific proteins, TPD enables the profound elimination of pathogenic drivers, thereby circumventing drug resistance associated with conventional small‐molecule inhibitors. Consequently, TPD holds immense promise for treating malignancies, neurodegenerative disorders, and inflammatory diseases [235, 236, 237].

Due to the structural complexity, multisubunit organization, and paucity of well‐defined druggable pockets within KMT2 family members, direct degraders of KMT2 proteins remain scarce. Accordingly, recent efforts have focused on targeted degradation of critical cofactors within KMT2 complexes to achieve functional inactivation of KMT2‐dependent transcriptional programs. Based on the WDR5 WIN‐site inhibitor OICR‐9429, VHL (Von Hippel–Lindau)‐recruiting WDR5 PROTACs (including MS33 and MS67) were developed to induce efficient WDR5 degradation. MS67 exhibited superior transcriptional suppression of WDR5‐regulated genes, reduced chromatin association of KMT2 complex components, and more potent antiproliferative activity compared with conventional WDR5 PPI inhibitors [238, 239]. Moreover, MS40, a PROTAC that recruits cereblon, specifically targets and degrades WDR5 and the Ikaros zinc finger transcription factors IKZF1/3. Due to MS40‐induced WDR5 degradation, the KMT2A complex dissociates from chromatin, causing a decrease in H3K4me2 levels [240]. In KMT2‐r leukemia, recognition of histone acetylation marks by the YEATS domain of the fusion partner ENL is essential for the maintenance of AML [241, 242]. ENL‐targeting PROTACs (including SR‐1114 and Compound 69) rapidly degrade ENL, markedly downregulate oncogenic genes such as HOXA10 and FMS‐like tyrosine kinase 3 (FLT3), and exhibit antitumor activity in KMT2A‐r leukemia and in Wilms tumors harboring ENL mutations [243, 244]. Collectively, these studies highlight targeted degradation of nonenzymatic scaffolding and transcriptional regulatory components within KMT2 complexes as a powerful and precise therapeutic strategy for KMT2‐driven diseases.

#### 4.2.3 Transcriptional Program Inhibitors

##### 4.2.3.1 DOT1L Inhibitors

DOT1L is crucial for gene transcription regulation, cell cycle oversight, and DNA damage repair, with its dysfunction being closely tied to the initiation and advancement of various types of cancer [245, 246, 247]. In KMT2A‐r leukemia, KMT2A fusion proteins aberrantly recruit DOT1L to the promoter regions of oncogenes such as HOXA9 and MEIS1, leading to excessive methylation of H3K79. This, in turn, maintains the transcriptional program of leukemia stem cells and drives leukemia development [97, 248].

Various DOT1L inhibitors have been developed so far [249, 250, 251]. Pinometostat (EPZ‐5676) became the first aminonucleoside DOT1L inhibitor to enter clinical trials, demonstrating extremely high affinity and selectivity [252, 253]. Clinical Phase I trials (NCT02141828, NCT01684150) showed that EPZ‐5676 is well tolerated, but its monotherapy efficacy is limited, with a low ORR and susceptibility to drug resistance and relapse [198, 199, 254].

In addition, studies have shown that DOT1L antagonizes SIRT1‐guided H3K9‐mediated transcriptional silencing, thereby contributing to the maintenance of KMT2 fusion target gene expression, which can be exploited to enhance the efficacy of DOT1L inhibition [255]. Accordingly, combined treatment with SIRT1 activators and DOT1L inhibitors exhibits enhanced antileukemic activity in KMT2‐rearranged leukemia cells. However, these agents generally exert slow pharmacodynamic effects and have demonstrated limited efficacy in clinical trials, which has restricted their further clinical development [256]. Current research efforts have shifted toward combination strategies to overcome therapeutic resistance.

##### 4.2.3.2 BET Inhibitors

BRD4, a prototypical epigenetic “reader” of the BET family, orchestrates transcriptional activation by recognizing chromatin acetylation marks [257, 258]. In KMT2A‐r leukemia, KMT2 fusion proteins collaborate with BET bromodomains to regulate RNA polymerase II‐mediated transcription. This interaction sustains the aberrant overexpression of critical oncogenic drivers, such as BCL2 and MYC, thereby maintaining the malignant phenotype [256, 259].

BET inhibitors have demonstrated potent antileukemic activity in KMT2A‐r AML. The small‐molecule inhibitor JQ1 induces cell‐cycle arrest and apoptosis, rendering KMT2A‐r infant leukemia and fusion‐driven leukemia cells highly sensitive to treatment [260]. Similarly, GSK1210151A (I‐BET151) exhibits significant efficacy against KMT2A fusion‐positive leukemia cell lines by inducing early cell‐cycle arrest and apoptosis [259]. To circumvent the narrow therapeutic window of first‐generation pan‐BET inhibitors, second‐generation molecules like ABBV‐744 and iBET‐BD2 have been developed to selectively target the BD2 domain, yielding enhanced potency and attenuated systemic toxicity [261, 262]. Despite the improved efficacy of BET inhibitors, acquired resistance has been observed during JQ1 treatment [263].

CRISPR‐based genetic screening in recent studies has revealed that SPOP is a significant gene responsible for BET inhibitor resistance in KMT2A‐r AML cell lines [259]. SPOP functions as a substrate adaptor of the cullin 3‐based E3 ubiquitin ligase complex and has been implicated in tumor progression in prostate, endometrial, and gastric cancers [264, 265, 266, 267, 268, 269]. Loss‐of‐function mutations in SPOP lead to stabilization of BRD4, thereby attenuating BET inhibitor‐mediated suppression of the MYC transcriptional program and conferring resistance to BET inhibition [270].

##### 4.2.3.3 LSD1 Inhibitors

Histone demethylase LSD1 catalyzes the demethylation of H3K4 and H3K9 sites [271]. Its dysregulated expression in various cancers promotes proliferation, hinders differentiation, and is associated with poor prognosis [272, 273, 274, 275, 276]. KMT2 and LSD1 help sustain balanced methylation at H3K4, an essential “histone code” for active gene transcription [277]. LSD1 maintains the differentiation block in KMT2A‐r AML and is essential for leukemic stem cell potential [278].

LSD1 inhibitors are now being tested in clinical trials, including tranylcypromine (TCP), iadademstat (ORY‐1001), bomedemstat (IMG‐7289), and pulrodemstat (CC‐90011) [271, 279, 280, 281]. In addition, covalent inhibition of LSD1 has been shown to derepress transcriptional programs associated with differentiation and apoptosis that are dysregulated in KMT2A‐r leukemia, thereby dismantling oncogenic transcriptional networks and restoring myeloid differentiation [282].

LSD1 inhibition promotes differentiation of KMT2A‐r AML cells primarily through disruption of the LSD1/CoREST/GFI1 repressor complex on chromatin [283]. Cyclopropylamine‐based LSD1 inhibitors exhibit potent and selective antileukemic activity against KMT2A‐r leukemias, with their efficacy closely correlating with the degree of LSD1 enzymatic inhibition [284]. GSK2879552, an orally bioavailable N‐alkylated derivative of TCP, induces a marked loss of clonogenic potential and promotes cellular differentiation, particularly in the KMT2‐translocated molecular subtype of AML [285].

Iadademstat is a powerful and highly selective covalent inhibitor of LSD1. It induces the accumulation of H3K4me2 at LSD1 target genes, promotes both molecular and morphological differentiation of primitive leukemia cells driven by KMT2 gene rearrangements, and reduces the proliferative capacity of leukemia stem cells in AML [279, 286, 287]. Preliminary data from a Phase I trial (EudraCT No. 2013‐002447‐29) underscored its favorable safety profile and early signals of clinical efficacy in R/R KMT2A‐r AML [200]. Moreover, preclinical research indicates that combining LSD1 inhibitors with all‐trans retinoic acid (ATRA) or mTOR inhibitors can boost therapeutic effectiveness [288, 289].

Beyond KMT2A‐driven leukemia, LSD1 inhibition may also be therapeutically relevant in tumors harboring alterations in other KMT2 family members. In medulloblastoma with heterozygous loss‐of‐function of KMT2D, KMT2D deficiency significantly diminished key enhancer‐associated histone modifications, including H3K4 monomethylation and H3K27 acetylation, as well as the H3K4 trimethylation mark, thereby promoting tumor progression. Combined treatment with the LSD1 inhibitor and OXPHOS inhibitor metformin significantly reduced the tumorigenicity of of tumor cells [290].

Collectively, LSD1 inhibition displays antitumor activity in various cancer models and enhances cancer cell differentiation, where combinatorial inhibition strategies produce synergistic effects in vitro and in vivo.

##### 4.2.3.4 Histone Deacetylase inhibitors

Histone deacetylases (HDACs) regulate chromatin architecture by catalyzing the deacetylation of lysine residues on histones, thereby modulating transcriptional accessibility and gene expression [291]. HDACs are frequently overexpressed in solid tumors, such as gastric and colorectal cancers, as well as in hematologic malignancies, including leukemia [292, 293, 294, 295], and can drive chromatin remodeling and aberrant oncogene expression through interactions with KMT2 fusion partners [296].

Knocking down Class IIA HDAC isoforms (HDAC4, HDAC5, and HDAC7) in KMT2A‐r ALL cells leads to cell cycle arrest and induces apoptosis. The HDAC4/5‐selective small molecule inhibitor LMK‐235 also effectively suppresses the proliferation of KMT2A‐r ALL cells [297]. A number of HDAC inhibitors have received approval for treating blood cancers, including vorinostat (SAHA), romidepsin (FK‐228), belinostat (PXD‐101), and panobinostat (LBH‐589) [298, 299]. Although not developed specifically for KMT2‐driven disease, these agents provide a clinical framework supporting the therapeutic relevance of HDAC inhibition in epigenetically dysregulated malignancies. A novel hydroxamate‐based HDAC inhibitor I1 (4‐(4‐(1H‐indol‐3‐yl)butanamido)‐N‐hydroxybenzamide) was reported to suppress cell proliferation and induce G0/G1 cell cycle arrest in KMT2A‐r acute leukemia models. Importantly, I1 promoted differentiation of KMT2A‐r leukemia cells and exhibited comparable or, in specific experimental settings, higher HDAC inhibitory activity than SAHA, supporting its potential as an epigenetic therapeutic candidate [300]. In addition, the pan‐HDAC inhibitor panobinostat (LBH589) reduces H2B ubiquitination by inhibiting the RNF20/RNF40/WAC E3 ligase complex and demonstrates significant in vivo antileukemic activity in PDX models of infant KMT2A‐r ALL [301].

Beyond KMT2A‐r leukemia, HDAC inhibition also shows therapeutic relevance in malignancies driven by alterations in other KMT2 family members. KMT2D ranks as one of the most commonly mutated genes in both follicular lymphoma and DLBCL [302, 303]. In B lymphocytes, KMT2D mutations interfere with H3K4 methylation, affecting the JAK–STAT, Toll‐like receptor, and B‐cell receptor signaling pathways, thereby promoting malignant proliferation [304, 305]. HDAC inhibitors can compensate for the histone acetylation defects caused by EP300/CREBBP mutations, rendering tumor cells more sensitive to SAHA [306, 307]. Combination therapy with HDAC inhibitors and decitabine synergistically suppresses proliferation and induces apoptosis in DLBCL cells [308, 309]. In addition, mutations in histone‐modifying genes, such as KMT2D, are associated with decreased progression‐free survival and better responsiveness to the HDAC inhibitor tucidinostat (chidamide) in patients diagnosed with peripheral T‐cell lymphoma [310]. In KMT2D‐mutant T‐cell lymphoma models, cotreatment with the chidamide and the HMA decitabine enhances the interaction between KMT2D and the transcription factor PU.1, modulates H3K4me3‐associated signaling pathways, sensitizes lymphoma cells to chidamide, and significantly delays tumor growth in xenograft models [310, 311, 312, 313].

### 4.3 Synergistic Therapeutic Strategies

Synergistic therapeutic strategies aim to overcome the limitations of single‐agent therapy by combining drugs with different mechanisms of action, thereby enhancing antitumor efficacy and delaying or overcoming treatment resistance. Current synergistic strategies centered on KMT2‐dependent transcriptional programs are primarily focused on three directions: first, pairing KMT2‐focused drugs with epigenetic agents such as inhibitors of DNA methyltransferase, LSD1 inhibitors, or BET inhibitors to synergistically remodel chromatin accessibility and transcriptional programs, restoring cell differentiation and homeostatic regulation; second, using in combination with traditional chemotherapy or molecular targeted drugs (such as BCL‐2 or FLT3 inhibitors) to synergistically inhibit abnormal proliferation through multiple pathways and induce programmed cell death, thereby delaying or overcoming acquired resistance; third, integrating with immunotherapy strategies, leveraging the genomic instability and immune‐activated microenvironment shaped by KMT2 family mutations, to synergistically enhance antitumor immune responses through the combination of epigenetic drugs and ICIs. The aforementioned synergistic modalities have demonstrated synergistic potentiating potential in preclinical and early clinical trials, providing new theoretical basis and practical directions for precision therapy of KMT2 family‐related diseases.

#### 4.3.1 Epigenetic‐Based Combination Therapies

While monotherapies targeting the KMT2 family have demonstrated preliminary efficacy, their clinical impact is frequently constrained by limited long‐term durability and the emergence of therapeutic resistance [314, 315]. Accumulating evidence indicates that KMT2‐driven tumors depend on the coordinated activity of multiple epigenetic modifiers to sustain aberrant transcriptional programs [316]. Consequently, combination strategies integrating KMT2‐targeted agents with epigenetic therapies are considered an important approach to enhance therapeutic efficacy and prolong the duration of response [317].

HMAs, including azacitidine, decitabine, and zebularine, reduce DNA hypermethylation and induce apoptosis in KMT2‐r ALL cells [318, 319, 320]. MEK inhibitors inhibit RAS‐mutant KMT2A‐r infant ALL and enhance efficacy when combined with decitabine [321, 322]. Short‐term high‐dose exposure to decitabine combined with cytarabine significantly enhances the suppression of KMT2A‐r ALL cells in vitro [320]. Moreover, in preclinical models of KMT2A‐r AML, chidamide works together with the Menin–KMT2A interaction inhibitor MI‐3 to reduce cell viability both in vitro and in vivo [323].

Clinical studies have demonstrated that the HDAC inhibitor vorinostat and the proteasome inhibitor bortezomib exhibit significant synergistic effects in KMT2A‐rearranged leukemia. A combination therapy further incorporating mitoxantrone achieved a complete remission rate of 80% and an overall response rate of 90% in patients [324]. The Phase I/II SAVE trial (NCT05360160) evaluated an all‑oral triplet regimen of ASTX727 with revumenib and venetoclax in patients with AML or mixed phenotype acute leukemia with myeloid features, demonstrating a high response rate and a notable degree of MRD negativity [325, 326]. Similarly, in the KOMET‐007 study (NCT05735184), the combination of ziftomenib with venetoclax and azacitidine demonstrated encouraging clinical activity in patients with KMT2A‐r AML, with a favorable safety profile and no ziftomenib‐associated QTc prolongation [206].

Beyond their role in targeting KMT2A‑driven transcriptional programs, KDM5 inhibitors also represent a promising strategy for KMT2D mutational lymphomas. In preclinical models, KDM5 inhibition elevates promoter‐associated H3K4me3 levels, In KMT2D‐mutant DLBCL cell lines such as SU‐DHL‐6 and OCI‐LY‐18, KDM5 inhibition induces marked cytotoxicity in vitro and inhibits tumor growth in SU‐DHL‐6 xenografts in vivo, supporting its potential as a targeted approach for KMT2D‐mutant lymphomas [327].

#### 4.3.2 Chemotherapy and Targeted Therapies

##### 4.3.2.1 Menin Inhibitors Plus Conventional Chemotherapy

Intensive chemotherapy, typified by the “7+3” regimen (7 days of cytarabine + 3 days of daunorubicin), has long been the standard treatment for AML; however, its applicability in older patients is limited by poor tolerability and high treatment‐related mortality [328, 329, 330]. Menin inhibitors have demonstrated the ability to induce remissions in heavily pretreated populations in clinical trials, with a more manageable safety profile and effective control of adverse events such as DS [331]. Nevertheless, acquired resistance following MI monotherapy remains a major clinical challenge [332].

Clinical evidence supports the benefit of combining MIs with conventional chemotherapy. In the KOMET‐007 trial (NCT05735184), the combination of ziftomenib with 7+3 induction chemotherapy resulted in high composite complete remission (CRc) rates, achieving 93% in NPM1‐m and 89% in KMT2A‐r AML patients, alongside favorable MRD‐negativity results and a tolerable safety profile without additive myelosuppression [333, 334]. Another clinical study (NCT05453903) reported that bleximenib combined with “7+3” achieves ORRs near 93% and CR/CRh 86% in newly diagnosed patients with NPM1 or KMT2A alterations, with manageable safety and without notable QTc prolongation or DS [209]. Preliminary clinical evidence further suggests that combination chemotherapy may reduce the incidence of MI‐associated DS, while adverse effects such as QTc prolongation appear to be more controllable [335]. In addition, the pediatric and adolescent ITCC‐101/APAL2020K trial (NCT06376162) similarly confirmed the favorable safety and tolerability of ziftomenib in combination with the FLA regimen [208]. Collectively, these findings indicate that combining menin inhibitors with chemotherapy can significantly improve clinical outcomes in patients with AML.

##### 4.3.2.2 Menin Inhibitors Combined with BCL‐2 Inhibition

BCL‐2 proteins bind to the BH3 domains of proapoptotic proteins, which helps cancer cells survive and resist chemotherapy [336, 337]. Leukemia cells harboring KMT2A‐r are highly sensitive to BCL‐2 inhibition. Venetoclax, the first approved BCL‐2 inhibitor, mimics the BH3 domain to disrupt the interaction between BCL‐2 and proapoptotic proteins, thereby activating the mitochondrial apoptotic pathway, and has been approved for the treatment of AML patients who are ineligible for intensive chemotherapy [338, 339, 340, 341]. Menin inhibitors downregulate BCL‑2 and synergize with venetoclax in KMT2A‑r leukemia [342]. Moreover, using the menin inhibitor MI‐503 alongside venetoclax reduces the expression of the hypoxia pathway's key factor HIF‐1α and its downstream protein HDAC9, thereby boosting antileukemic effects [343].

The BAMT trial (NCT03013998) assessed a triplet regimen consisting of a menin inhibitor combined with venetoclax and an HMA, demonstrating high response rates and favorable tolerability [203]. This triplet strategy has now been extended from adult patients to adolescents and pediatric populations. A Phase I study, such as ZiVA (NCT06397027), is currently evaluating its safety and preliminary efficacy, with evidence suggesting that this combination can effectively overcome resistance to menin inhibitors [207]. Mechanistic studies indicate that loss of the polycomb repressive complex 1.1 (PRC1.1) represents a key mechanism of resistance to menin inhibitors, and AML cells with PRC1.1 deficiency exhibit markedly increased sensitivity to venetoclax, providing a potential precision‐based strategy to overcome therapeutic resistance [344, 345, 346].

##### 4.3.2.3 Dual Targeting of Menin and FLT3 Signaling

FLT3 mutations are enriched in KMT2A‐r ALL, resulting in FLT3 activation or overexpression [347]. This co‐occurrence provides a strong rationale for dual targeting of the KMT2A–menin axis and FLT3 signaling [348].

Preclinical studies have demonstrated marked synergistic effects between menin inhibitors and FLT3 inhibitors in KMT2A‐r or NPM1‐m leukemia, effectively suppressing FLT3 phosphorylation and downstream gene expression, thereby inhibiting leukemic cell proliferation and delaying the emergence of resistance [348]. The observed synergy has now advanced into clinical evaluation. In the Phase I KOMET‐008 trial (NCT06222580), ziftomenib is being assessed in combination with the FLT3 inhibitor gilteritinib for patients with relapsed/refractory FLT3‐mutated AML harboring KMT2A‐r or NPM1‐m. Preliminary results indicate a favorable safety profile and promising antileukemic activity [349]. Earlier clinical efforts also support this approach: the Children's Oncology Group trial AALL0631 (NCT00557193) demonstrated that in infants with KMT2A‐r ALL, the combination of the FLT3 inhibitor lestaurtinib with chemotherapy yielded a significant correlation between strong FLT3 pharmacodynamic inhibition and improved long‐term event‐free survival [211].

#### 4.3.3 Integration of Immunotherapy in KMT2‐Altered Tumors

Cancer immunotherapy has achieved substantial clinical benefit across multiple malignancies by activating antitumor immune responses, including cytokine‐based therapies, cancer vaccines, ICIs, and adoptive cell therapy (ACT) [350, 351, 352, 353]. However, the response to ICIs in clinical settings varies, and there is an urgent need for predictive biomarkers [354]. Loss‐of‐function mutations in KMT2 family genes are associated with clinical outcomes in patients receiving anti PD‐1/PD‐L1 therapy, particularly in solid tumors and selected hematologic malignancies [159, 355, 356].

CAR T‐cell therapy is used in blood cancers, a major form of ACT, targeting CD19 or CD22, has induced high remission rates in relapsed/refractory B‐cell ALL (B‐ALL). In infants with KMT2A‐r ALL, CD19‐directed CAR T‐cell therapies (including CTL019, huCART19, and tisagenlecleucel) have achieved durable remissions and significantly improved 2‐year relapse‐free survival [357]. However, selective immune pressure from CAR T cells can trigger a myeloid lineage switch, resulting in loss of B‐lineage antigens such as CD19 and subsequent immune escape, a resistance mechanism predominantly observed in KMT2A‐r ALL [358, 359, 360, 361]. Systematic dependency analyses identified FLT3 as a surface protein specifically upregulated and genetically essential in KMT2A‐r B‐ALL [362]. Accordingly, FLT3‐directed CAR T cells have emerged as a rational combination immunotherapy strategy for both KMT2A‐r ALL and FLT3‐mutant AML [363]. Preclinical studies have demonstrated that FLT3CART and bispecific CD19×FLT3 CAR T cells exhibit potent in vitro and in vivo antileukemic activity against FLT3‐mutant AML and KMT2A‐r ALL, with robust leukemia clearance in PDX models of KMT2A‐r ALL. Using PiggyBac transposon‐engineered dual‐target CAR T cells, additional studies further showed preserved cytotoxicity in CD19‐negative immune escape models and CD19‐knockout (CD19‐KO) KMT2A‐r B‐ALL relapse models, supporting dual targeting as a strategy to overcome antigen escape [364]. Beyond conventional CAR T‐cell platforms, a recent study have developed off‐the‐shelf dual‐target CAR‐iNKT cells (CD19×CD133) for high‐risk KMT2A‐r ALL. This approach exploits PROM1/CD133 high expression to simultaneously target B‐lineage antigens and KMT2A‐driven antigens. Compared with mono‐specific or conventional CAR‐T cells, dual CAR‐iNKT cells demonstrate superior performance in vitro and in vivo, effectively removing medullary and leptomeningeal leukemia and ensuring enduring remissions [365].

## 5 Conclusions and Perspectives

The KMT2 gene family plays a pivotal role in epigenetic regulation by catalyzing the methylation of histone H3K4, crucial for gene expression, cell differentiation, and tissue homeostasis. As demonstrated in this review, dysregulation of the KMT2 family serves as a core driver in multiple disease spectra, with its members participating in pathogenic processes through distinct molecular mechanisms. The oncogenic fusion protein derived from KMT2A‐r exemplifies how chromatin regulatory factors are hijacked to sustain leukemia stem cell status. Meanwhile, the frequent loss‐of‐function mutations of KMT2C/D in solid tumors reveal their critical roles in metabolic homeostasis and DNA repair. Furthermore, germline mutations highlight the dose‐sensitive and indispensable functions of this family member in neurodevelopment.

Despite these significant advances, critical challenges and unresolved issues remain, highlighting key directions for future research: first, while the oncogenic role of KMT2A in leukemia is well established, its function in solid tumors such as lung cancer remains unclear. Similarly, KMT2D predominantly exerts tumor‐suppressive effects, but its deletion in specific contexts (e.g., KRAS‐mutated LUAD) can create metabolic vulnerability. This profound context‐dependent nature may stem from cell‐type‐specific transcriptional circuits, comutation backgrounds, and metabolic states. At present, the development of treatments targeting the KMT2 family shows a significant imbalance: protein interaction inhibitors (such as menin inhibitors) for KMT2A‐r leukemia have achieved clinical breakthroughs, while for the more common KMT2C/D loss‐of‐function in solid tumors, no directly corresponding targeted therapies have emerged yet. This disparity is rooted in their distinct pathogenic mechanisms: the former is a “gain of function,” which is easier to design blocking drugs for; the latter is a “loss of function,” which is difficult to directly restore.

To address this challenge, future research should go beyond the traditional strategy of directly targeting the KMT2C/D proteins, and systematically explore the indirect vulnerabilities exposed by their functional absence. This can be achieved by advancing in the following three interrelated aspects: first, through functional compensation and epigenetic reprogramming, small molecule regulators capable of restoring the activity and transcriptional program of key enhancers downstream of KMT2C/D are developed to reverse the oncogenic cell state. Second, using the high tumor mutation burden and “immune heat” phenotype associated with KMT2C/D mutations as biomarkers, they are combined with ICIs (such as anti‐PD‐1/PD‐L1 therapies); future research should delve deeper into the specific mechanisms driving immune infiltration to design more precise immune combination regimens. Finally, based on the principle of synthetic lethality, target genes or pathways with synthetic lethality relationships to KMT2C/D deletion are systematically screened, such as the glycolysis‐dependent enhancement found in LUAD with KMT2D deletion, which provides a basis for developing novel targeted combination therapies.

Based on this, it is necessary to actively expand and optimize the combined treatment strategies. Initial studies suggest that menin inhibitors and venetoclax work together synergistically, FLT3 inhibitors or epigenetic drugs. Future clinical trials can explore the combined application of menin inhibitors with drugs targeting the PRC1.1 complex or SWI/SNF chromatin remodeling factors, in terms of the resistance mechanisms. For cancers with KMT2C/D deficiency, reasonable combined treatment regimens can include epigenetic regulators (such as BET, HDAC, or EZH2 inhibitors) combined with immunotherapy, PARP inhibitors, or metabolic intervention, to address their specific transcriptional and metabolic dependencies. Moreover, for NDDs caused by germline mutations in the KMT2 family, developing treatments that can correct epigenetic dysregulation or restore gene expression is also an important direction in future translational medicine.

In summary, the KMT2 research has delved into mechanisms and targeted therapies at the genetic level. Future efforts should address its functional complexity by employing novel technologies to elucidate its context‐dependent molecular basis, develop innovative regulatory therapies, and complete rigorous clinical translation validation.

## Author Contributions

Yan Liu, Jian Zhang, and Ajing Xu supervised the research and provided overall guidance during the preparation of the manuscript. Qiu Wang, Zunjie Bo, and Ya Zhang were responsible for drafting and revising the manuscript. Yun Chen, Zhiyu Wang, and Shuangmei Tong designed the figures and tables. All authors reviewed and approved the final version of the manuscript.

## Funding

This work was funded by the National Natural Science Foundation of China (Grant Nos. 82173891) and the Shanghai Municipal Health Commission (Grant No. 20244Z0002),Two Hundred Talents Program of Shanghai Jiao Tong University School of Medicine(Grant No. 20250521).

## Ethics Statement

The authors have nothing to report.

## Conflicts of Interest

The authors declare no conflicts of interest.

## Data Availability

The authors have nothing to report.
